# Conventional and genetic associations of BMI with major vascular and non-vascular disease incidence and mortality in a relatively lean Chinese population: U-shaped relationship revisited

**DOI:** 10.1093/ije/dyae125

**Published:** 2024-10-09

**Authors:** Andri Iona, Fiona Bragg, Zammy Fairhurst-Hunter, Iona Y Millwood, Neil Wright, Kuang Lin, Ling Yang, Huaidong Du, Yiping Chen, Pei Pei, Liang Cheng, Dan Schmidt, Daniel Avery, Canqing Yu, Jun Lv, Robert Clarke, Robin Walters, Liming Li, Sarah Parish, Zhengming Chen, China Kadoorie Biobank Collaborative Group, Junshi Chen, Zhengming Chen, Robert Clarke, Rory Collins, Yu Guo, Liming Li, Chen Wang, Jun Lv, Richard Peto, Robin Walters, Daniel Avery, Derrick Bennett, Ruth Boxall, Ka Hung Chan, Yumei Chang, Yiping Chen, Zhengming Chen, Johnathan Clarke, Robert Clarke, Huaidong Du, Ahmed Edris Mohamed, Zammy Fairhurst-Hunter, Hannah Fry, Simon Gilbert, Alex Hacker, Mike Hill, Michael Holmes, Pek Kei Im, Andri Iona, Maria Kakkoura, Christiana Kartsonaki, Kuang Lin, Mohsen Mazidi, Iona Millwood, Sam Morris, Qunhua Nie, Alfred Pozarickij, Paul Ryder, Saredo Said, Sam Sansome, Dan Schmidt, Paul Sherliker, Rajani Sohoni, Becky Stevens, Iain Turnbull, Robin Walters, Lin Wang, Neil Wright, Ling Yang, Xiaoming Yang, Pang Yao, Yu Guo, Xiao Han, Can Hou, Qingmei Xia, Chao Liu, Jun Lv, Pei Pei, Canqing Yu, Naying Chen, Duo Liu, Zhenzhu Tang, Ningyu Chen, Qilian Jiang, Jian Lan, Mingqiang Li, Yun Liu, Fanwen Meng, Jinhuai Meng, Rong Pan, Yulu Qin, Ping Wang, Sisi Wang, Liuping Wei, Liyuan Zhou, Caixia Dong, Pengfei Ge, Xiaolan Ren, Zhongxiao Li, Enke Mao, Tao Wang, Hui Zhang, Xi Zhang, Jinyan Chen, Ximin Hu, Xiaohuan Wang, Zhendong Guo, Huimei Li, Yilei Li, Min Weng, Shukuan Wu, Shichun Yan, Mingyuan Zou, Xue Zhou, Ziyan Guo, Quan Kang, Yanjie Li, Bo Yu, Qinai Xu, Liang Chang, Lei Fan, Shixian Feng, Ding Zhang, Gang Zhou, Yulian Gao, Tianyou He, Pan He, Chen Hu, Huarong Sun, Xukui Zhang, Biyun Chen, Zhongxi Fu, Yuelong Huang, Huilin Liu, Qiaohua Xu, Li Yin, Huajun Long, Xin Xu, Hao Zhang, Libo Zhang, Jian Su, Ran Tao, Ming Wu, Jie Yang, Jinyi Zhou, Yonglin Zhou, Yihe Hu, Yujie Hua, Jianrong Jin Fang Liu, Jingchao Liu, Yan Lu, Liangcai Ma, Aiyu Tang, Jun Zhang, Liang Cheng, Ranran Du, Ruqin Gao, Feifei Li, Shanpeng Li, Yongmei Liu, Feng Ning, Zengchang Pang, Xiaohui Sun, Xiaocao Tian, Shaojie Wang, Yaoming Zhai, Hua Zhang, Wei Hou, Silu Lv, Junzheng Wang, Xiaofang Chen, Xianping Wu, Ningmei Zhang, Weiwei Zhou, Xiaofang Chen, Jianguo Li, Jiaqiu Liu, Guojin Luo, Qiang Sun, Xunfu Zhong, Weiwei Gong, Ruying Hu, Hao Wang, Meng Wan, Min Yu, Lingli Chen, Qijun Gu, Dongxia Pan, Chunmei Wang, Kaixu Xie, Xiaoyi Zhang, Shuya Li, Haiqiang Qin, Yongjun Wang, Qiling Chen, Jihua Wang, Xiaojia Sun, Lei Wang, Xun Wang, Liming Zhang, Shanshan Zhou, Hongyuan Chen, Li Chen, Haiyan Gou, Weizhi Wang, Yanmei Zhu, Yulan Zhu, Ning Zhang, Xin Cheng, Qiang Dong, Yi Dong, Kun Fang, Yiting Mao, Yu An, Peiling Chen, Yinghua Chen, Zhihong Liu, Lihua Zhang Xiaohong Chen, Naixin Jv, Xiaojiu Li, Liyang Liu, Yun Lu, Xiaona Xing, Shihao You, Xiaoli Cheng, Chaojun Gua, Jinping Jiang, Jingyi Liu, Shumei Ma, Xuefeng Yang, Xiaomo Du, Jian Xu, Xuecheng Yang, Xiaodi Zhao, Zilong Hao, Ming Liu, Deren Wang, Xiaoting Li, Lili Hui, Zhanling Liao, Feng Liu, Chunning Feng, Dejiang Ji, Fengxia Qu, Wenwen Yuan, Xin Fu, Jing Ding, Peng Du, Lirong Jin, Yueshi Mao, Xin Wang

**Affiliations:** Clinical Trial Service Unit & Epidemiological Studies Unit (CTSU), Nuffield Department of Population Health, University of Oxford, Oxford, UK; Clinical Trial Service Unit & Epidemiological Studies Unit (CTSU), Nuffield Department of Population Health, University of Oxford, Oxford, UK; Health Data Research UK Oxford, University of Oxford, Oxford, UK; Clinical Trial Service Unit & Epidemiological Studies Unit (CTSU), Nuffield Department of Population Health, University of Oxford, Oxford, UK; Clinical Trial Service Unit & Epidemiological Studies Unit (CTSU), Nuffield Department of Population Health, University of Oxford, Oxford, UK; Clinical Trial Service Unit & Epidemiological Studies Unit (CTSU), Nuffield Department of Population Health, University of Oxford, Oxford, UK; Clinical Trial Service Unit & Epidemiological Studies Unit (CTSU), Nuffield Department of Population Health, University of Oxford, Oxford, UK; Clinical Trial Service Unit & Epidemiological Studies Unit (CTSU), Nuffield Department of Population Health, University of Oxford, Oxford, UK; Clinical Trial Service Unit & Epidemiological Studies Unit (CTSU), Nuffield Department of Population Health, University of Oxford, Oxford, UK; Clinical Trial Service Unit & Epidemiological Studies Unit (CTSU), Nuffield Department of Population Health, University of Oxford, Oxford, UK; Peking University Center for Public Health and Epidemic Preparedness & Response, Beijing, China; Qingdao Shinan District Centre for Disease Control and Prevention, Shinan District, Qingdao, China; Clinical Trial Service Unit & Epidemiological Studies Unit (CTSU), Nuffield Department of Population Health, University of Oxford, Oxford, UK; Clinical Trial Service Unit & Epidemiological Studies Unit (CTSU), Nuffield Department of Population Health, University of Oxford, Oxford, UK; Peking University Center for Public Health and Epidemic Preparedness & Response, Beijing, China; Department of Epidemiology and Biostatistics, School of Public Health, Peking University Health Science Center, Beijing, China; Key Laboratory of Epidemiology of Major Diseases (Peking University), Ministry of Education, Beijing, China; Peking University Center for Public Health and Epidemic Preparedness & Response, Beijing, China; Department of Epidemiology and Biostatistics, School of Public Health, Peking University Health Science Center, Beijing, China; Key Laboratory of Epidemiology of Major Diseases (Peking University), Ministry of Education, Beijing, China; Clinical Trial Service Unit & Epidemiological Studies Unit (CTSU), Nuffield Department of Population Health, University of Oxford, Oxford, UK; Clinical Trial Service Unit & Epidemiological Studies Unit (CTSU), Nuffield Department of Population Health, University of Oxford, Oxford, UK; Peking University Center for Public Health and Epidemic Preparedness & Response, Beijing, China; Department of Epidemiology and Biostatistics, School of Public Health, Peking University Health Science Center, Beijing, China; Key Laboratory of Epidemiology of Major Diseases (Peking University), Ministry of Education, Beijing, China; Clinical Trial Service Unit & Epidemiological Studies Unit (CTSU), Nuffield Department of Population Health, University of Oxford, Oxford, UK; Clinical Trial Service Unit & Epidemiological Studies Unit (CTSU), Nuffield Department of Population Health, University of Oxford, Oxford, UK

**Keywords:** Body mass index, Mendelian randomization, cardiovascular disease, stroke

## Abstract

**Background:**

Higher body mass index (BMI) is associated with higher incidence of cardiovascular and some non-cardiovascular diseases (CVDs/non-CVDs). However, uncertainty remains about its associations with mortality, particularly at lower BMI levels.

**Methods:**

The prospective China Kadoorie Biobank recruited >512 000 adults aged 30–79 years in 2004–08 and genotyped a random subset of 76 000 participants. In conventional and Mendelian randomization (MR) analyses, Cox regression yielded adjusted hazard ratios (HRs) associating measured and genetically predicted BMI levels with incident risks of major vascular events (MVEs; conventional/MR 68 431/23 621), ischaemic heart disease (IHD; 50 698/12 177), ischaemic stroke (IS; 42 427/11 897) and intracerebral haemorrhage (ICH; 7644/4712), and with mortality risks of CVD (15 427/6781), non-CVD (26 915/4355) and all causes (42 342/6784), recorded during ∼12 years of follow-up.

**Results:**

Overall, the mean BMI was 23.8 (standard deviation: 3.2) kg/m^2^ and 13% had BMIs of <20 kg/m^2^. Measured and genetically predicted BMI showed positive log-linear associations with MVE, IHD and IS, but a shallower positive association with ICH in conventional analyses. Adjusted HRs per 5 kg/m^2^ higher genetically predicted BMI were 1.50 (95% CI 1.41–1.58), 1.49 (1.38–1.61), 1.42 (1.31–1.54) and 1.64 (1.58–1.69) for MVE, IHD, IS and ICH, respectively. These were stronger than associations in conventional analyses [1.21 (1.20–1.23), 1.28 (1.26–1.29), 1.31 (1.29–1.33) and 1.14 (1.10–1.18), respectively]. At BMIs of ≥20 kg/m^2^, there were stronger positive log-linear associations of BMI with CVD, non-CVD and all-cause mortality in MR than in conventional analyses.

**Conclusions:**

Among relatively lean Chinese adults, higher genetically predicted BMI was associated with higher risks of incident CVDs. Excess mortality risks at lower BMI in conventional analyses are likely not causal and may reflect residual reverse causality.

Key Messages

- In this large, relatively lean [mean body mass index (BMI) 23.8 kg/m^2^] Chinese population, there were stronger positive log-linear associations of BMI with incident major vascular events, ischaemic heart disease, ischaemic stroke and intracerebral haemorrhage in Mendelian randomization than in conventional analyses.
- For cardiovascular disease (CVD), non-CVD and all-cause mortality, there were positive log-linear associations at BMIs of >20 kg/m^2^, with stronger genetic than conventional associations.
- Higher BMI levels were likely causally associated with higher risks of incidence of and mortality from CVD and CVD types across a wide BMI distribution.

## Background

Worldwide, excess adiposity affects ∼2 billion adults and its prevalence continues to rise.^1^ Previous observational studies have found positive log-linear associations of adiposity, typically assessed using body mass index (BMI), with risks of incident cardiovascular diseases (CVDs), with modestly stronger associations with ischaemic heart disease (IHD) than with ischaemic stroke (IS).^2–9^ For haemorrhagic stroke, which has particularly high rates in China, the reported evidence was conflicting, with positive^3^^,^^4^^,^^6^^,^^8^^,^^10^ and inverse^7^ log-linear associations. For CVD and all-cause mortality, however, most studies found U- or J-shaped associations, which persisted after extensive attempts to control for reverse causality and confounding.^11–16^ BMI levels associated with the lowest mortality risk in these studies varied greatly, from 25 to 35 kg/m^2^.^12^^,^^13^^,^^17–21^

Reliable assessment of the shape, strength and causality of associations of BMI with disease incidence and mortality can inform strategies for tackling obesity and obesity-related disease burden. Mendelian randomization (MR) can help to minimize effects of reverse causality and confounding biases.^22^^,^^23^ Previous linear MR studies in Western populations have reported positive associations of genetically predicted BMI with IHD incidence that are stronger than associations of measured BMI.^24^ For stroke, the strength of both conventional and genetic associations of BMI were strongest for intracerebral haemorrhage (ICH) and large artery stroke, with no apparent associations with cardio-embolic stroke.^25^^,^^26^ For all-cause and CVD mortality, given the U- and J-shaped associations in observational studies, emerging non-linear MR methods have been used in Western populations, which have reported mixed findings, including J-shaped^27^^,^^28^ and positive log-linear associations.^29^ Inconsistencies between studies may reflect differences in the methodologies employed^30–32^ or in the populations studied. For example, few participants (∼5%) in these Western population studies had BMIs of <20 kg/m^2^, resulting in imprecise causal estimates at low BMI.^27–29^ This contrasts with higher proportions (∼15–20%) in East Asian populations.^11^^,^^27–29^ However, no previous non-linear MR studies have explored the relationship of adiposity with mortality in an East Asian population. Moreover, to date, no studies have simultaneously examined the shape of conventional and genetic associations of BMI with risks of major CVD incidence and mortality and non-CVD mortality in the same study population.

The present report examines conventional and genetic associations of BMI with major diseases in the prospective China Kadoorie Biobank (CKB) study. The main objectives are to: (i) assess and compare the strength and shape of associations of measured and genetically predicted BMI with risks of incident major vascular events (MVEs); and (ii) examine and compare conventional and genetic associations of BMI with all-cause, CVD and non-CVD mortality.

## Methods

### Study population

CKB is a prospective study of 512 869 participants aged 30–79 years, recruited during 2004–08 from 10 diverse areas in China (Supplementary Text, available as Supplementary data at *IJE* online).^33^^,^^34^ Anthropometric measurements were recorded at recruitment with participants wearing light clothing and no shoes. BMI was calculated as weight in kilograms divided by the square of the height in metres.

For MR analyses, data were available for ∼100 000 participants, including 75 920 participants who were randomly selected from the total cohort (Supplementary Figure S1, available as Supplementary data at *IJE* online).^35^ A trans-ethnic BMI genetic score (GS) was used for the main MR analyses, which was not associated with potential confounders, with a UK Biobank (UKB)-driven BMI–GS used in sensitivity analyses (Supplementary Text; Supplementary Table S2, available as Supplementary data at *IJE* online).^36^

Information on incident disease events was obtained through linkage to health insurance records and disease registries (Supplementary Text, available as Supplementary data at *IJE* online). Mortality was monitored via China’s Disease Surveillance Points system. The main outcomes of interest included incidence of MVEs, IHD, IS and ICH, in addition to CVD, non-CVD and all-cause mortality (Supplementary Table S3, available as Supplementary data at *IJE* online).

All participants provided written informed consent.

### Statistical analyses

In the conventional analyses, individuals with missing BMI data (*n* = 2) and self-reported diagnosed CVD (*n* = 23 129) at recruitment were excluded (Supplementary Figure S1, available as Supplementary data at *IJE* online). The prevalence or mean values of baseline characteristics were calculated overall and across BMI categories (cut points: 20.0, 25.0 and 30.0 kg/m^2^), standardized to the age (5-year groups), sex and study area structure of the study population. Cox proportional-hazards models were used to estimate hazard ratios (HRs) for disease outcomes, stratified by age-at-risk, sex and study area, and adjusted for education and lifestyle factors.^37^ In sensitivity analyses, we further excluded: (i) individuals with any self-reported major chronic diseases or poor self-rated health at recruitment; (ii) ever smokers; and (iii) outcomes that occurred during the first 3 years of follow-up (which also showed no strong evidence of departure from the proportional-hazards assumption).

In genetic analyses, MR estimates were calculated by using the ratio method, dividing the estimated outcome–GS associations by estimates of the BMI–GS association. Outcome–GS associations were estimated by using Cox proportional-hazards models stratified for age-at-risk (5-year groups), sex and study area, adjusted for 11 genetic (national) principal components (PCs).^35^ BMI–GS associations were estimated using linear regression adjusted for age (5-year groups), sex, study area and 11 PCs.

We also undertook non-linear MR analyses using doubly ranked stratification.^32^^,^^38^ We first stratified participants into 10 pre-strata based on levels of the BMI–GS, followed by further stratification based on levels of baseline BMI within each pre-stratum. We then calculated MR estimates for each of the 10 strata by using the ratio method.^23^ The denominator of the ratio was estimated in the random genotyping subset to avoid potential biases that could have resulted from including a large number of selected CVD cases. The numerator was estimated in the random genotyping subset for all-cause and non-CVD mortality, and among the random genotyping subset and additional CVD cases for CVD outcomes. The stratum-specific estimate of the BMI–GS association was used as the denominator. These genetic estimates were meta-regressed against the mean BMI in each stratum by using the fractional polynomial function of two degrees. In these analyses, the reference was set to the mean BMI and the CIs were estimated by using standard errors from the meta-regression.^23^

In sensitivity analyses, we separately utilized a denominator from the random genotyping subset and additional CVD cases, excluded those with self-reported or doctor-diagnosed CVD and additionally adjusted for variables that were included in the conventional analyses. We additionally examined BMI–CVD associations by using the residual method^35^ and investigated BMI–GS associations with age and sex as potential negative controls.

All analyses were performed by using SAS (version 9.3) and R (version 4.1.2, packages ‘ggplot2’, ‘ckbplotr’, ‘nlmr’ and ‘SUMnlmr’).

## Results

Among 489 593 participants who were included in the conventional analyses, the mean baseline age was 52.0 (SD 10.6) years and the mean BMI was 23.7 (3.2) kg/m^2^. The proportions of ever regular smoking and regular alcohol drinking were higher in men than in women, and men with BMIs of <20 kg/m^2^ were more likely to smoke and less likely to drink alcohol (Table 1). BMI was strongly positively associated with systolic blood pressure (SBP) and prevalence of hypertension, diabetes and CVD, but showed U-shaped relationships with prevalence of chronic obstructive pulmonary disease and self-rated poor health. These associations were similar among 75 981 participants included in MR analyses (Table 1 and Supplementary Table S4, available as Supplementary data at *IJE* online).

**Table 1.** Characteristics of study participants by measured BMI at baseline

Characteristics^a^	BMI (kg/m^2^)				**All** (*n*=489 593)	**Random genotyping subset** (*n*=75 981)
	**<20** (*n*=66 699)	**20.0 to <25.0** (*n*=267 603)	**25.0 to <30.0** (*n*=136 751)	**≥30.0** (*n*=18 540)		
**Age and socio-economic factors**						
Age [years (SD)]^b^	52.7 (12.7)	51.0 (10.4)	51.5 (10.2)	51.5 (10.2)	52.0 (10.6)	51.6 (10.4)
Women (%)^c^	59.1	57.7	60.6	71.1	59.3	59.8
Urban (%)^d^	31.6	40.3	52.8	58.8	43.1	48.3
≥6 Years of education (%)	48.1	49.6	49.6	48.1	52.1	51.0
Household income >20 000 yuan (%)	11.6	54.3	30.2	3.83	42.8	41.1
**Anthropometry and blood pressure [mean (SD)]**						
BMI (kg/m^2^)	18.8 (1.2)	22.6 (1.4)	26.8 (1.4)	31.8 (2.2)	23.7 (3.2)	23.8 (3.2)
Waist circumference (cm)	68.3 (5.9)	77.7 (5.9)	88.1 (6.2)	99.0 (8.9)	80.3 (9.0)	80.3 (9.0)
Waist-to-hip ratio	0.82 (0.07)	0.87 (0.06)	0.92 (0.06)	0.96 (0.08)	0.88 (0.06)	0.88 (0.06)
Percentage body fat	18.9 (3.7)	26.2 (4.0)	33.5 (4.6)	40.1 (6.9)	27.9 (6.4)	27.9 (6.4)
Systolic blood pressure (mmHg)	123 (22)	129 (19)	136 (20)	143 (26)	131 (20)	131 (19)
Heart rate (bpm)	79 (14)	78 (12)	80 (13)	82 (16)	79 (11)	79 (12)
**Lifestyle factors**						
Ever regular smoker (%)						
Men	79.8	75.4	70.8	70.8	74.3	73.8
Women	5.1	3.3	2.9	3.5	3.2	3.3
Regular alcohol consumption (%)						
Men	32.9	37.2	36.9	36.4	37.2	38.6
Women	2.4	2.5	2.6	2.2	2.3	2.6
Physical activity [MET-h/day (SD)]	21.8 (13.6)	21.9 (11.8)	20.8 (12.4)	19.5 (14.9)	21.1 (11.7)	21.5 (11.8)
**Self-reported doctor-diagnosed medical history (%)**						
Diabetes^e^	3.5	4.9	7.8	11.3	6.2	6.2
Cardiovascular diseases	2.8	3.9	5.6	7.1	4.1	4.1
Uncontrolled hypertension^f^	2.7	6.2	12.7	20.4	9.0	11.8
Chronic respiratory disease	15.3	8.8	6.8	6.8	9.1	9.1
Chronic liver disease	1.5	1.2	1.1	1.1	1.2	1.3
Chronic kidney disease	1.3	1.4	1.7	1.8	1.5	1.5
Cancer^g^	0.3	0.1	0.1	0.2	0.2	0.2
Self-rated poor health	17.8	9.9	9.9	13.4	13.5	10.3

BMI, body mass index; DBP, diastolic blood pressure; MET, metabolic equivalent of task; SBP, systolic blood pressure.

a Adjusted for age, sex and study area, unless specified otherwise.

b Adjusted for sex and study area.

c Adjusted for age and study area.

d Adjusted for age and sex.

e Self-reported or screen-detected diabetes.

f Self-reported hypertension and SBP ≥140 mmHg or DBP ≥90mmHg.

g Self-reported cancer and on treatment at baseline.

**Figure 1.**
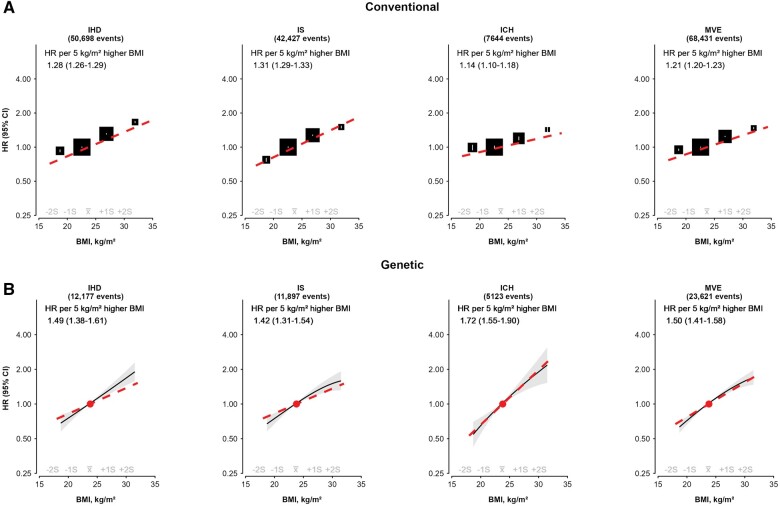
(a) Conventional and (b) genetic associations of BMI with incident risks of ischaemic heart disease, ischaemic stroke, intracerebral haemorrhage and major vascular events. The slopes of the associations of BMI with each outcome are shown as hazard ratios (HRs; 95% CIs) per 5-kg/m^2^ higher BMI. The HRs in conventional analyses for BMI were stratified by age-at-risk (5-year age groups), sex and study area (10 groups), and adjusted for education (5 groups), smoking (4 groups), alcohol intake (5 groups) and physical activity [metabolic equivalent of task (MET) hours per day] and are shown as squares, with 95% CIs shown as vertical lines. In MR analyses, the fractional polynomial method was used to estimate the relationship between BMIs and outcomes [stratified by age-at-risk (5-year age groups), sex and study area (10 groups), and adjusted for 11 genetic PCs]. The reference was set to mean BMI (23.8 kg/m^2^). The 95% CIs in MR analyses are represented by the shaded patterns. BMI, body mass index; HR, hazard ratio; ICH, intracerebral haemorrhage; IHD, ischaemic heart disease; IS, ischaemic stroke; MR, Mendelian randomization; MVE, major vascular events; PCs, principal components

After 12 years of follow-up, 68 431 participants experienced an MVE, including 23 621 in the genetic subset (12 177 IHD, 11 897 IS and 5123 ICH) (Table 2, Figure 1 and Supplementary Table S2, available as Supplementary data at *IJE* online). In both conventional and MR analyses, there were positive and approximately log-linear associations of BMI with risks of incident MVE, IHD and IS throughout the BMI range that was examined (17.0–34.0 kg/m^2^). HRs per 5-kg/m^2^ higher BMI were stronger in genetic than conventional analyses for MVE [1.50 (95% CI 1.41–1.58) vs 1.21 (1.20–1.23); *P_heterogeneity_* <0.001] and IHD [1.49 (1.38–1.61) vs 1.28 (1.26–1.29); *P_heterogeneity_* <0.001], but were equally strong for IS [1.42 (1.31–1.54) vs 1.31 (1.29–1.33); *P_heterogeneity_* = 0.05]. For ICH, there was a weak positive association in conventional analyses [1.14 (1.10–1.18)] but a strong positive, apparently log-linear association [1.72 (1.55–1.90)] in MR analyses.

**Table 2.** Genetic associations of BMI with risks of incident cardiovascular diseases

BMI strata^a^	BMI (kg/m^2^)		GS–BMI^b^Estimate (SE)	No. of events	GS–outcome^c^Estimate (SE)	LACE^d^Estimate (SE)
	Range	Mean				
**Major vascular events**						
Strata 1	12.6–26.3	18.7	2.07 (0.064)	2383	0.19 (0.076)	0.094 (0.037)
Strata 2	15.2–27.0	20.6	2.31 (0.056)	2104	0.24 (0.081)	0.104 (0.035)
Strata 3	17.1–27.4	21.5	2.49 (0.053)	2207	0.18 (0.078)	0.072 (0.032)
Strata 4	17.7–28.3	22.4	2.70 (0.052)	2146	0.21 (0.080)	0.078 (0.030)
Strata 5	18.9–29.0	23.2	2.81 (0.053)	2144	0.18 (0.080)	0.063 (0.029)
Strata 6	24.0–19.2	24.0	2.90 (0.055)	2251	0.50 (0.078)	0.173 (0.027)
Strata 7	24.9–19.7	24.9	3.06 (0.058)	2369	0.28 (0.077)	0.090 (0.025)
Strata 8	25.9–20.7	25.9	3.25 (0.063)	2488	0.20 (0.075)	0.061 (0.023)
Strata 9	27.2–21.5	27.2	3.34 (0.074)	2686	0.14 (0.073)	0.041 (0.022)
Strata 10	22.4–47.1	31.5	3.72 (0.107)	2843	0.20 (0.069)	0.053 (0.019)
Test for *heterogeneity*			*X* ^2^=447.6, *P* <0.001		*X* ^2^=15.5, *P* =0.077	*X* ^2^=18.8, *P* =0.027
**Ischaemic heart disease**						
Strata 1	12.6–26.3	18.7	2.07 (0.064)	1070	0.13 (0.133)	0.062 (0.055)
Strata 2	15.2–27.0	20.6	2.31 (0.056)	861	0.25 (0.253)	0.110 (0.055)
Strata 3	17.1–27.4	21.5	2.49 (0.053)	958	0.08 (0.083)	0.034 (0.048)
Strata 4	17.7–28.3	22.4	2.70 (0.052)	982	0.32 (0.323)	0.117 (0.043)
Strata 5	18.9–29.0	23.2	2.81 (0.053)	1091	0.17 (0.173)	0.061 (0.040)
Strata 6	24.0–19.2	24.0	2.90 (0.055)	1177	0.27 (0.273)	0.091 (0.037)
Strata 7	24.9–19.7	24.9	3.06 (0.058)	1255	0.21 (0.213)	0.069 (0.034)
Strata 8	25.9–20.7	25.9	3.25 (0.063)	1387	0.41 (0.413)	0.126 (0.031)
Strata 9	27.2–21.5	27.2	3.34 (0.074)	1564	0.13 (0.133)	0.039 (0.028)
Strata 10	22.4–47.1	31.5	3.72 (0.107)	1832	0.33 (0.333)	0.089 (0.024)
Test for *heterogeneity*			*X* ^2^=447.6, *P<* 0.001		*X* ^2^ = 8.9, *P*=0.438	*X* ^2^ = 7.0, *P* = 0.633
**Ischaemic stroke**						
Strata 1	12.6–26.3	18.7	2.07 (0.064)	874	0.17 (0.125)	0.082 (0.060)
Strata 2	15.2–27.0	20.6	2.31 (0.056)	871	0.29 (0.127)	0.126 (0.055)
Strata 3	17.1–27.4	21.5	2.49 (0.053)	952	0.06 (0.119)	0.023 (0.048)
Strata 4	17.7–28.3	22.4	2.70 (0.052)	1059	0.24 (0.114)	0.088 (0.042)
Strata 5	18.9–29.0	23.2	2.81 (0.053)	1141	0.03 (0.111)	0.012 (0.039)
Strata 6	24.0–19.2	24.0	2.90 (0.055)	1205	0.25 (0.108)	0.088 (0.037)
Strata 7	24.9–19.7	24.9	3.06 (0.058)	1250	0.29 (0.106)	0.096 (0.035)
Strata 8	25.9–20.7	25.9	3.25 (0.063)	1373	0.37 (0.103)	0.115 (0.032)
Strata 9	27.2–21.5	27.2	3.34 (0.074)	1542	0.18 (0.096)	0.053 (0.029)
Strata 10	22.4–47.1	31.5	3.72 (0.107)	1630	0.09 (0.091)	0.024 (0.024)
Test for *heterogeneity*			*X* ^2^ = 447.6, *P* < 0.001		*X* ^2^ = 9.8, *P* = 0.367	*X* ^2^ = 10.9, *P* = 0.284
**Intracerebral haemorrhage**						
Strata 1	12.6–26.3	18.7	2.07 (0.064)	710	0.33 (0.138)	0.161 (0.066)
Strata 2	15.2–27.0	20.6	2.31 (0.056)	552	0.13 (0.159)	0.054 (0.069)
Strata 3	17.1–27.4	21.5	2.49 (0.053)	549	0.34 (0.158)	0.138 (0.063)
Strata 4	17.7–28.3	22.4	2.70 (0.052)	501	0.25 (0.165)	0.093 (0.061)
Strata 5	18.9–29.0	23.2	2.81 (0.053)	511	0.47 (0.163)	0.169 (0.058)
Strata 6	24.0–19.2	24.0	2.90 (0.055)	468	0.39 (0.174)	0.133 (0.060)
Strata 7	24.9–19.7	24.9	3.06 (0.058)	425	0.42 (0.183)	0.137 (0.060)
Strata 8	25.9–20.7	25.9	3.25 (0.063)	467	0.11 (0.173)	0.033 (0.053)
Strata 9	27.2–21.5	27.2	3.34 (0.074)	462	0.36 (0.177)	0.108 (0.053)
Strata 10	22.4–47.1	31.5	3.72 (0.107)	478	0.35 (0.170)	0.093 (0.046)
Test for *heterogeneity*			*X* ^2^ = 447.6, *P* < 0.001		*X* ^2^ = 4.6, *P* = 0.868	*X* ^2^ = 5.1, *P* = 0.824

BMI, body mass index; CVD, cardiovascular disease; GS, genetic score; HR, hazard ratio; LACE, localized average causal effect; SE, standard error.

a BMI strata refer to doubly ranked strata.

b The estimates of BMI in kg/m^2^ per 1 GS SD (from the association of BMI–GS and BMI) across the BMI strata in the random genotyping subset.

c The log HR per 1 GS SD from Cox proportional-hazards models (stratified by age-at-risk, sex and study area) to estimate the association of BMI–GS with outcomes across BMI strata. This analysis was conducted among the random genotyping subset and CVD cases combined.

d The LACE estimates (log HR per average GS 1 kg/m^2^) are calculated as the ratio of the log HR from the GS–outcome association and the estimate from the BMI–GS association across BMI strata.

**Figure 2.**
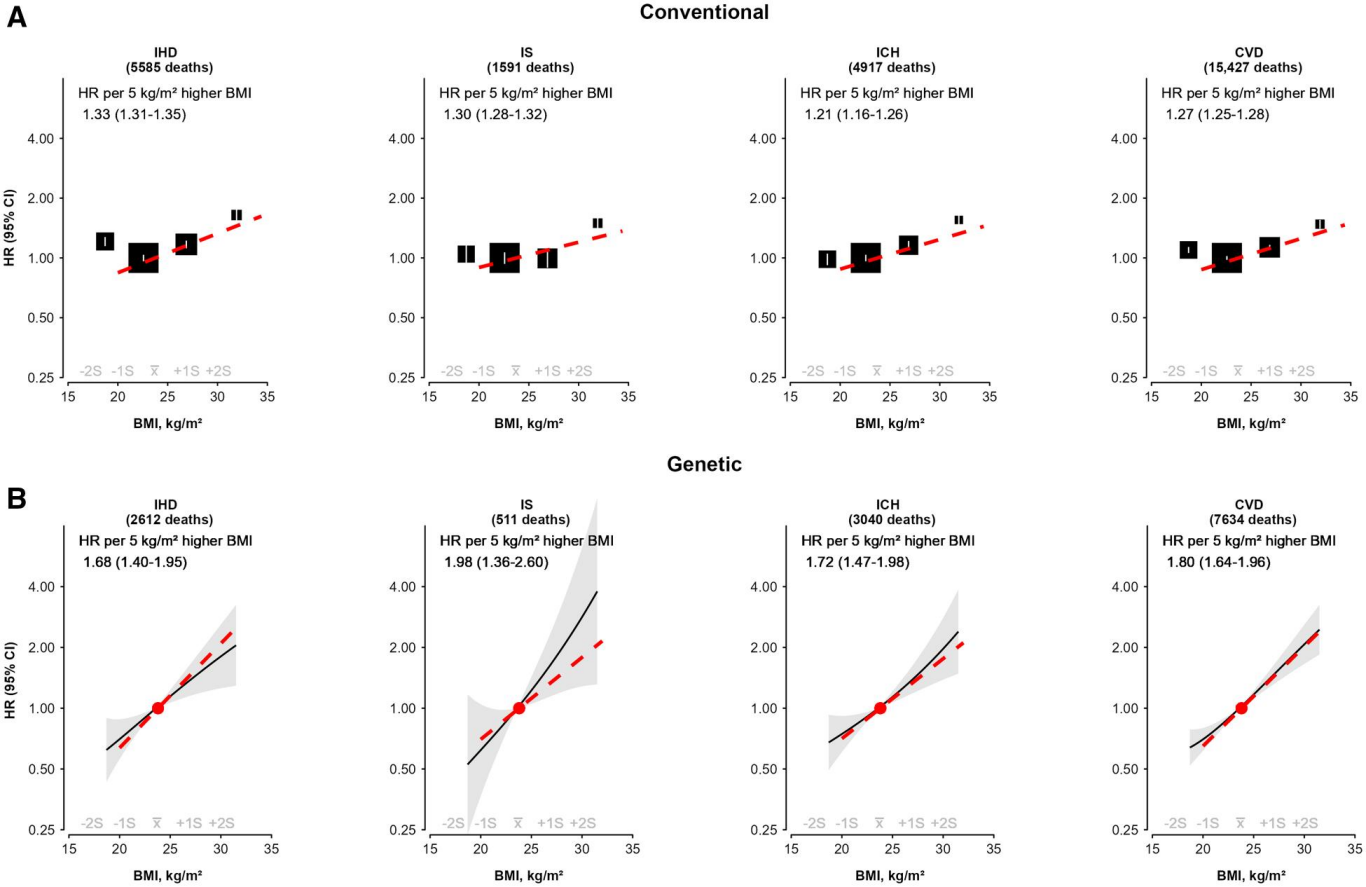
(a) Conventional and (b) genetic associations of BMI with mortality risks of ischaemic heart disease, ischaemic stroke, intracerebral haemorrhage and CVD. Conventions as in Figure 1. The slopes of the associations of BMI with each outcome per 5-kg/m^2^ higher BMI were estimated at BMI ≥20.0 kg/m^2^. BMI, body mass index; CVD, cardiovascular disease; HR, hazard ratio; ICH, intracerebral haemorrhage; IHD, ischaemic heart disease; IS, ischaemic stroke

For mortality outcomes, both conventional and MR analyses showed positive log-linear associations of BMI with IHD, IS, ICH and overall CVD mortality at BMIs of ≥20 kg/m^2^, with no clear evidence of association at lower BMI levels, possibly reflecting small event numbers (Figure 2, Supplementary Table S6 and Supplementary Figure S7, available as Supplementary data at *IJE* online). At BMIs of ≥20.0 kg/m^2^, each 5-kg/m^2^ higher genetically predicted BMI was associated with HRs of 1.68 (1.40–1.95), 1.98 (1.36–2.60), 1.72 (1.47–1.98) and 1.80 (1.64–1.96) for IHD, IS, ICH and overall CVD mortality, respectively, which again were stronger (*P_heterogeneity_* <0.01) than corresponding HRs in conventional analyses (e.g. 1.21 for ICH and 1.33 for IHD).

For non-CVD (*n* = 26 915) and all-cause (*n* = 42 342) mortality, conventional analyses showed approximately U-shaped associations with BMI, with lowest risks at 25.0–29.9 kg/m^2^ (Supplementary Figure S2, available as Supplementary data at *IJE* online). These results were unaltered after excluding unhealthy individuals and, additionally, ever smokers, or the first 3 years of follow-up (Supplementary Figures S3–S5, available as Supplementary data at *IJE* online). In MR analyses, however, there were strong positive, log-linear associations with non-CVD (*n* = 4355) and all-cause (*n* = 6784) mortality at BMIs of ≥20 kg/m^2^ (Supplementary Figure S2, available as Supplementary data at *IJE* online), with each 5-kg/m^2^ increment associated with HRs of 1.59 (1.37–1.81) and 1.72 (1.55–1.89), respectively.

**Figure 3.**
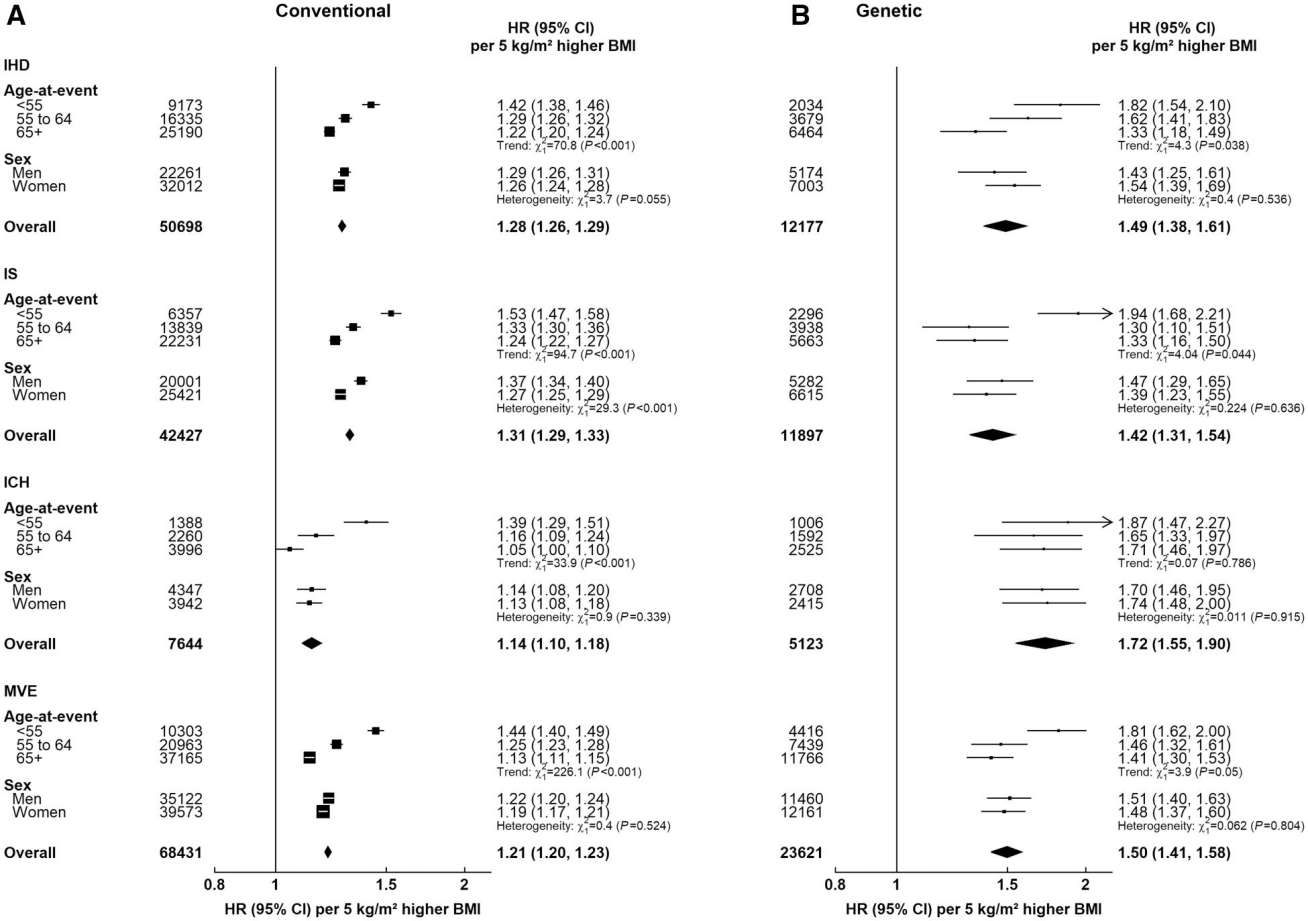
Adjusted HRs for incident cardiovascular diseases per 5-kg/m^2^ higher BMI by age and sex in (a) conventional and (b) MR analyses. The HRs are shown as squares and 95% CIs are shown as horizontal lines. The chi-square and *P*-values are shown for heterogeneity or linear trend between sex- and age-specific groups, respectively. Conventional HRs for BMI were stratified by age-at-risk (5-year age groups), sex and study area (10 groups), and adjusted for education (5 groups), smoking (4 groups), alcohol intake (5 groups) and physical activity [metabolic equivalent of task (MET) hours per day]. MR HRs were estimated by using the ratio method [stratified by age-at-risk (5-year age groups), sex and study area (10 groups), and adjusted for 11 genetic PCs]. BMI, body mass index; HR, hazard ratio; ICH, intracerebral haemorrhage; IHD, ischaemic heart disease; IS, ischaemic stroke; MR, Mendelian randomization; MVE, major vascular events; PCs, principal components

In both conventional and MR analyses, there were clear trends of higher HRs at younger ages for IHD and IS (*P_trend_* <0.05; Figure 3) whereas, for ICH and MVE, the trend in MR analyses appeared less clear. However, in both MR and conventional analyses, there were no age-related trends for CVD, non-CVD and all-cause mortality outcomes (Supplementary Figures S1–S6, available as Supplementary data at *IJE* online). For incident IS, the HRs were greater in men than in women (*P_heterogeneity_* <0.001) in conventional analyses but not in MR analyses whereas, for incident IHD, ICH and MVE and mortality outcomes, there were no clear sex differences.

MR findings did not alter after the exclusion of participants with prior CVD at baseline (Supplementary Figure S8, available as Supplementary data at *IJE* online) or when the denominator (i.e. the association of the GS with the BMI) was estimated among the random genotyping subset and CVD cases (Supplementary Figure S9, available as Supplementary data at *IJE* online) or after additional adjustment for variables that were included in the conventional analyses (Supplementary Figure S10, available as Supplementary data at *IJE* online). The doubly ranked and residual methods showed similar BMI–CVD associations (Supplementary Figure S11, available as Supplementary data at *IJE* online). There was evidence of heterogeneity in the effect of BMI on sex and age across BMI strata (Supplementary Figure S12, available as Supplementary data at *IJE* online). However, when linear MR was used, genetically predicted BMI was not associated with sex [estimate 0.16 (SE 0.60) per 5-kg/m^2^ higher BMI, *P*-value = 0.79] or age [OR 0.98 (95% CI 0.90–1.11), *P*-value = 0.99].

## Discussion

This is the first large East Asian population study to assess and compare conventional and genetic associations of BMI with risks of CVD incidence and mortality. In this relatively lean Chinese population, there were positive log-linear associations of measured and genetically predicted BMI with risks of incident MVE, IHD, IS and ICH, with risk estimates that were stronger in MR than in conventional analyses, especially for ICH. MR analyses also demonstrated positive log-linear associations of BMI with CVD, non-CVD and all-cause mortality, at least at BMI levels of ≥20.0 kg/m^2^, in contrast with more modest associations in conventional analyses. Thus, the parallel conventional and MR analyses support the causal relevance of adiposity for a range of major CVD outcomes and highlight the limitations of conventional analyses, especially for mortality outcomes, to control fully for residual biases.

Previous prospective studies and meta-analyses of such studies have provided consistent evidence on the shape and strength of conventional associations of BMI with incident CVD.^2–4^^,^^6^^,^^7^^,^^10^^,^^39^^,^^40^ In the Emerging Risk Factors Collaboration, which involved predominantly Western populations, there were positive log-linear associations of BMI with risks of IHD (5259 events) and IS (2431 events).^2^ Within the BMI range of 20.0–38.0 kg/m^2^, each 5-kg/m^2^ higher BMI was associated with 32% and 22% higher IHD and stroke risks, respectively. The present study included a 9-fold greater number of IHD and 18-fold greater number of IS cases than this previous meta-analysis and showed similar excess risks per 5-kg/m^2^ higher BMI for incident IHD (26%) and IS (29%) in Chinese adults, with no evidence of a threshold within the BMI range of 17.0–34.0 kg/m^2^ (Supplementary Figure S7, available as Supplementary data at *IJE* online).

For incident ICH, previous studies have reported conflicting results, with log-linear positive^3^^,^^4^^,^^6^^,^^10^ or inverse^7^ associations with BMI, or a positive log-linear association only within the overweight/obese range.^39^^,^^40^ Most of these previous studies were conducted prior to the widespread use of neuro-imaging for the reliable diagnosis of stroke and stroke types. Moreover, they were constrained by relatively small numbers of ICH cases (e.g. ∼2800 in the largest study).^7^ In contrast, the present study included a much larger number of well-characterized (>90% confirmed by neuro-imaging) ICH cases (*n* = 7644).^41^ Despite this, we found only weak positive associations of BMI with incident ICH in conventional analyses, which is unexpected given the strong positive linear associations of BMI with SBP and of SBP with incident ICH in our study population.^9^ These disparities are too extreme to be accounted for by chance, known confounding or reverse causality. However, our MR analyses demonstrated a strong positive log-linear association of BMI with ICH, suggesting that other unmeasured or unknown risk factors that are associated with low BMI may be offsetting the protective effects of the lower blood pressure that is associated with lower BMI in conventional analyses.

Although many previous MR analyses have provided BMI-associated risk estimates for incident CVD and the main CVD types,^24–26^^,^^42^ none has properly examined the shape of these associations across different ranges of BMI distribution. In the CARDIoGRAMplusC4D study, each 5-kg/m^2^ higher genetically predicted BMI was associated with a 40% higher risk of incident IHD (66 842 cases), similarly to our estimates (35%).^24^ For IS, CARDIoGRAMplusC4D^24^ (12 389 events) and MEGASTROKE^26^ (60 341 events) reported 9–14% higher risk per SD (4.6 kg/m^2^) increment in genetically predicted BMI, which is much weaker than the association observed herein (34%). These differences might reflect differences in study design, populations and statistical analyses or varying proportions of IS subtypes. Likewise, MEGASTROKE reported a weaker positive association with ICH [*n* = 1545; 1.26 (0.90–1.75)]^26^ than was observed in CKB [1.72 (1.55–1.90)]. MEGASTROKE estimates for IS were derived from two-sample MR using summary statistics for BMI from UKB and the Health and Retirement Study (*n* ∼ 680 000) and for ICH from the International Stroke Genetic Consortium (1481 controls, 1545 ICH).^26^ However, our study used a one-sample MR approach that incorporated individual participant data from CKB and a trans-ethnic BMI–GS. Moreover, our findings did not alter when we used a UKB BMI–GS (Supplementary Figure S8, available as Supplementary data at *IJE* online).

For all-cause and CVD mortality, previous prospective studies typically reported U- or J-shaped associations with adiposity,^11^^,^^43–48^ with the lowest mortality risk usually observed in the so-called ‘normal’ BMI range.^11^^,^^43^^,^^44^^,^^46^ However, it is unclear whether these associations, particularly at lower BMI levels, reflect a true effect of BMI on mortality or were driven by uncontrolled reverse causality or residual confounding. Although traditional MR analyses are less prone to such biases, they assume a linear relationship between exposure and outcome, masking any non-linear associations.^22^^,^^23^ More recent non-linear MR methods seek to elucidate the shape of such associations. An earlier non-linear MR analysis employed the residual method among ∼400 000 European ancestry UKB participants, of whom ∼11 000 died. In contrast with the log-linear positive association observed in the present study, that study reported a J-shaped association of genetically predicted BMI with all-cause mortality, with the lowest risk at BMIs of ∼23 kg/m^2^.^27^ This observation was initially supported by findings from a separate study based on UKB (∼10 000 deaths) and the Trøndelag Health Study (∼12 000 deaths).^28^ Again, using the residual method, a J-shaped association was observed, with the lowest mortality risks at BMI levels of 22–23 kg/m^2^ in the HUNT Study and 25 kg/m^2^ in UKB and a positive log-linear association above this. In UKB, each 5-kg/m^2^ higher genetically derived BMI was associated with a 16% higher all-cause mortality risk [1.16 (0.95–1.40)] within the BMI range examined (18–45 kg/m^2^)^27^ compared with a HR of 1.72 (1.55–1.89) observed in the present study. There was a similar weak J-shaped association with CVD mortality (2145 deaths) in UKB. The differences in strengths of genetic associations at both lower and higher levels of BMI between our study and previous Western population studies^27^^,^^28^ may reflect differences in disease prevalence and in the proportion of disease types (e.g. stroke vs IHD, IS vs ICH), in addition to the proportion of participants with BMIs of <20 kg/m^2^ (∼15% in CKB vs ∼3% in UKB) affecting the power to detect associations. A subsequent report of the same HUNT and UKB data using a different (doubly ranked) method observed a different association between genetically predicted BMI and all-cause mortality, particularly in UKB, in which there was a positive log-linear association at BMI levels of >25 kg/m^2^ but a relatively flat association at lower BMIs, with statistical tests providing little evidence for non-linearity.^29^ Our findings from two non-linear MR methods are reasonably consistent with each other and with those from doubly ranked MR analyses in UKB,^29^ suggesting increasing mortality risks at higher BMI levels. The discrepant findings between residual and doubly ranked methods in UKB may reflect a predominance of older, frailer individuals among the underweight strata in this population whereas, in CKB, most lean people tended to be healthy. However, the inconsistent findings observed between non-linear MR approaches in some studies and the unexpected ‘non-null’ negative control associations in the present study and elsewhere highlight the need for caution in interpreting the findings, which may be susceptible to currently incompletely understood methodological biases.^49–53^

The present study has several strengths, including the large sample size, availability of reliably measured BMI and well-characterized cause-specific mortality and incident disease outcomes and completeness of follow-up. Moreover, the relatively lean population enabled investigation of the associations at lower BMI levels. Furthermore, conventional and genetic associations were simultaneously examined and compared in the same population. Finally, we used a trans-ethnic BMI–GS for the main analyses and a BMI–GS using UKB single-nucleotide polymorphisms for sensitivity analyses, and applied non-linear MR to assess the shape and strength of likely causal associations. However, the study has limitations. First, the MR analyses were based on a subset of the CKB population, which limited the power of subgroup analyses. Second, this report focuses only on BMI and does not investigate the relative importance of different adiposity measures (e.g. waist circumference). Third, as described, the non-linear MR method is still evolving and, as a result, the findings of those analyses should be interpreted with caution. Fourth, whereas this study includes more individuals with low BMIs, enhancing reliability, the absence of evidence for increased mortality risk at lower BMI levels could stem from either a genuine lack of association or insufficient power to detect an association. Fifth, the non-Gaussian distribution of BMI may adversely influence estimates of the BMI–GS association with BMI.^27^^,^^28^ Sixth, it did not explore the effect of potential mediators (e.g. blood pressure) on the genetic associations of BMI. Lastly, the relatively lean study population limited our ability to explore associations at higher BMI levels in detail.

In conclusion, this large study of Chinese adults demonstrated likely causal positive log-linear associations of BMI with risks of incident CVD and mortality at BMIs of ≥20.0 kg/m^2^. In conventional analyses, lower BMI levels were associated with higher risks of death, potentially reflecting uncontrolled biases. The findings highlight the importance of weight management strategies to reduce the adverse effects of high BMIs on CVD incidence and mortality.

## Ethics approval

Ethics approval was obtained from the Oxford University Tropical Research Ethics Committee and the Chinese Center for Disease Control and Prevention Ethical Review Committee prior to commencing the study.

## Supplementary Material

dyae125_Supplementary_Data

## Data Availability

Details of how to access data from the CKB study are available at www.ckbiobank.org or by contacting ckbiobank@ndph.ox.ac.uk. Custom code was used for the statistical analyses in this manuscript.
