# Supplementary material for: Conventional and genetic associations of BMI with major vascular and non-vascular disease incidence and mortality in a relatively lean Chinese population: U-shaped relationship revisited
Source: Int J Epidemiol. 2024 Oct 9;53(5):dyae125. doi: 10.1093/ije/dyae125 (PMC11464668; doi:10.1093/ije/dyae125)
Supplement: dyae125_Supplementary_Data [file dyae125_supplementary_data.docx]

Supplemental Online Content

**Conventional and genetic associations of BMI with major vascular and non-vascular disease incidence and mortality in a relatively lean Chinese population: U-shaped relationship revisited**

[Members of the China Kadoorie Biobank collaborative group 2](#_Toc175047082)

[Supplementary text 3](#_Toc175047083)

[Table S1. Reported conventional and genetic associations of BMI with risks of different CVDs in major published studies and meta-analyses 5](#_Toc175047084)

[Table S2. Baseline characteristics by tertiles of BMI genetic score in the random genotyping subset 6](#_Toc175047085)

[Table S3. ICD-10 codes for disease outcomes analysed 7](#_Toc175047086)

[Table S4. Characteristics of participants by measured BMI at baseline in the random genotyping subset 8](#_Toc175047087)

[Table S5. Conventional associations of BMI with risks of CVD incidence and CVD, non-CVD and all-cause mortality 9](#_Toc175047088)

[Table S6. Genetic associations of BMI with risks of CVD, non-CVD and all-cause mortality 10](#_Toc175047089)

[Figure S1. Flow diagram of participants included in (A) conventional and (B) genetic analyses 11](#_Toc175047090)

[Figure S2. (A) Conventional and (B) genetic associations of BMI with risks of non-CVD and all-cause mortality 12](#_Toc175047091)

[Figure S3. Conventional associations of BMI with risks of CVD, non-CVD and all-cause mortality in (A) healthy and (B) unhealthy participants 13](#_Toc175047092)

[Figure S4. Conventional associations of BMI with risks of CVD, non-CVD and all-cause mortality among healthy participants by smoking status 14](#_Toc175047093)

[Figure S5. Conventional associations of BMI with risks of CVD, non-CVD and all-cause mortality among healthy participants by years of follow-up 15](#_Toc175047094)

[Figure S6. Adjusted HRs for CVD and non-CVD mortality risks per 5 kg/m^2^ higher BMI at BMI ≥20.0 kg/m^2^ by age and sex in (A) conventional and (B) genetic analyses 16](#_Toc175047095)

[Figure S7. Conventional associations of BMI with risks of CVD (A) incidence and (B) mortality using seven BMI categories 17](#_Toc175047096)

[Figure S8. Genetic associations of BMI with risks of CVD (A) incidence and (B) mortality excluding participants with self-reported doctor diagnosed CVD at baseline 18](#_Toc175047097)

[Figure S9. Genetic associations of BMI with risks of CVD (A) incidence and (B) mortality estimating the denominator in the random genotyping subset only and in the random genotyping subset plus CVD cases 19](#_Toc175047098)

[Figure S10. Genetic associations of BMI with risks of CVD (A) incidence and (B) mortality additionally adjust for the variables included in the conventional analyses 20](#_Toc175047099)

[Figure S11. Genetic associations of BMI with risks of CVD (A) incidence and (B) mortality using the doubly ranked method vs. the residual method 21](#_Toc175047100)

[Figure S12. Genetic associations of BMI with age and sex doubly ranked vs. residual method 22](#_Toc175047101)

[Figure S13. Genetic associations of BMI with risks of CVD (A) incidence and (B) mortality using BMI genetic score with UKB SNPs and weights 23](#_Toc175047102)

[Supplementary references 24](#_Toc175047103)

#

# Members of the China Kadoorie Biobank collaborative group

**International Steering Committee:** Junshi Chen, Zhengming Chen (PI), Robert Clarke, Rory Collins, Yu Guo, Liming Li (PI), Chen Wang, Jun Lv, Richard Peto, Robin Walters.

**International Co-ordinating Centre, Oxford:** Daniel Avery, Derrick Bennett, Ruth Boxall, Ka Hung Chan, Yumei Chang, Yiping Chen, Zhengming Chen, Johnathan Clarke; Robert Clarke, Huaidong Du, Ahmed Edris Mohamed, Zammy Fairhurst-Hunter, Hannah Fry, Simon Gilbert, Alex Hacker, Mike Hill, Michael Holmes, Pek Kei Im, Andri Iona, Maria Kakkoura, Christiana Kartsonaki, Kuang Lin, Mohsen Mazidi, Iona Millwood, Sam Morris, Qunhua Nie, Alfred Pozarickij, Paul Ryder, Saredo Said, Sam Sansome, Dan Schmidt, Paul Sherliker, Rajani Sohoni, Becky Stevens, Iain Turnbull, Robin Walters, Lin Wang, Neil Wright, Ling Yang, Xiaoming Yang, Pang Yao.

**National Co-ordinating Centre, Beijing:** Yu Guo, Xiao Han, Can Hou, Qingmei Xia, Chao Liu, Jun Lv, Pei Pei, Canqing Yu.

**10 Regional Co-ordinating Centres: Guangxi** Provincial CDC: Naying Chen, Duo Liu, Zhenzhu Tang. Liuzhou CDC: Ningyu Chen, Qilian Jiang, Jian Lan, Mingqiang Li, Yun Liu, Fanwen Meng, Jinhuai Meng, Rong Pan, Yulu Qin, Ping Wang, Sisi Wang, Liuping Wei, Liyuan Zhou. **Gansu** Provincial CDC: Caixia Dong, Pengfei Ge, Xiaolan Ren. Maiji CDC: Zhongxiao Li, Enke Mao, Tao Wang, Hui Zhang, Xi Zhang. **Hainan** Provincial CDC: Jinyan Chen, Ximin Hu, Xiaohuan Wang. Meilan CDC: Zhendong Guo, Huimei Li, Yilei Li, Min Weng, Shukuan Wu. **Heilongjiang** Provincial CDC: Shichun Yan, Mingyuan Zou, Xue Zhou. Nangang CDC: Ziyan Guo, Quan Kang, Yanjie Li, Bo Yu, Qinai Xu. **Henan** Provincial CDC: Liang Chang, Lei Fan, Shixian Feng, Ding Zhang, Gang Zhou. Huixian CDC: Yulian Gao, Tianyou He, Pan He, Chen Hu, Huarong Sun, Xukui Zhang. **Hunan** Provincial CDC: Biyun Chen, Zhongxi Fu, Yuelong Huang, Huilin Liu, Qiaohua Xu, Li Yin. Liuyang CDC: Huajun Long, Xin Xu, Hao Zhang, Libo Zhang. **Jiangsu** Provincial CDC: Jian Su, Ran Tao, Ming Wu, Jie Yang, Jinyi Zhou, Yonglin Zhou. Suzhou CDC: Yihe Hu, Yujie Hua, Jianrong Jin Fang Liu, Jingchao Liu, Yan Lu, Liangcai Ma, Aiyu Tang, Jun Zhang. **Qingdao** Qingdao CDC: Liang Cheng, Ranran Du, Ruqin Gao, Feifei Li, Shanpeng Li, Yongmei Liu, Feng Ning, Zengchang Pang, Xiaohui Sun, Xiaocao Tian, Shaojie Wang, Yaoming Zhai, Hua Zhang, Licang CDC: Wei Hou, Silu Lv, Junzheng Wang. **Sichuan** Provincial CDC: Xiaofang Chen, Xianping Wu, Ningmei Zhang, Weiwei Zhou. Pengzhou CDC: Xiaofang Chen, Jianguo Li, Jiaqiu Liu, Guojin Luo, Qiang Sun, Xunfu Zhong. **Zhejiang** Provincial CDC: Weiwei Gong, Ruying Hu, Hao Wang,Meng Wan, Min Yu. Tongxiang CDC: Lingli Chen, Qijun Gu, Dongxia Pan，Chunmei Wang, Kaixu Xie, Xiaoyi Zhang.

**Beijing Tiantan Hospital,Capital Medical University** Shuya Li, Haiqiang Qin, Yongjun Wang, **Peking University People's Hospital** Qiling Chen, Jihua Wang, **The 1^st^ Affiliated Hospital of Harbin Medical University** Xiaojia Sun, Lei Wang, Xun Wang, Liming Zhang, Shanshan Zhou, **The 2^nd^ Affiliated Hospital of Harbin Medical University** Hongyuan Chen, Li Chen, Haiyan Gou, Weizhi Wang, Yanmei Zhu, Yulan Zhu, **The 2^nd^ Hospital of Hebei Medical University** Ning Zhang, **Huashan Hospital** Xin Cheng, Qiang Dong, Yi Dong, Kun Fang, Yiting Mao, **Jinling Hospital** Yu An, Peiling Chen, Yinghua Chen, Zhihong Liu, Lihua Zhang **The People's Hospital of Liaoning Province** Xiaohong Chen, Naixin Jv, Xiaojiu Li, Liyang Liu, Yun Lu, Xiaona Xing, **Qingdao Fuwai Cardiovascular Hospital** Shihao You, **Shengjing Hospital of China Medical University** Xiaoli Cheng, Chaojun Gua, Jinping Jiang, Jingyi Liu, Shumei Ma, **Shenyang Military General Hospital** Xuefeng Yang, **The First People's Hospital of Shenyang** Xiaomo Du, Jian Xu, Xuecheng Yang, Xiaodi Zhao, **West China Hospital, Sichuan University** Zilong Hao, Ming Liu, Deren Wang, **The Second Affiliated Hospital of Suzhou University** Xiaoting Li, **Suzhou Kowloon Hospital Shanghai Jiao Tong University School of Medicine** Lili Hui, Zhanling Liao, Feng Liu, **Qingdao Fuwai Cardiovascular Hospital** Chunning Feng, Dejiang Ji, Fengxia Qu, Wenwen Yuan, **The First Affiliated Hospital of Zhengzhou University** Xin Fu, **Zhongshan Hospital,** Jing Ding, Peng Du, Lirong Jin, Yueshi Mao, Xin Wang.

# Supplementary text

*Study Population*

Details of the China Kadoorie Biobank (CKB) design and methods have been reported previously.^1, 2^ Briefly, participants were recruited between 2004 and 2008 from 10 diverse areas (5 urban, 5 rural), chosen from China’s nationally representative Disease Surveillance Points (DSP) to ensure geographic and social diversity, as well as diversity in exposure and disease patterns. All 1,801,200 permanent, non-disabled residents aged 35-74 were invited to participate, and 512,869 were enrolled (including 12,665 just outside the target age range, making the actual range 30-79 years). Trained health workers administered laptop-based questionnaires at local study clinics, collecting information on sociodemographic and lifestyle factors, and medical history.

Anthropometric measurements were recorded with participants wearing light clothing but without shoes, and usually to the nearest 0.1 cm or 0.1 kg. Body mass index (BMI) was calculated as weight in kilograms (measured using a TANITA-TBF-300GS) divided by the square of the height in metres (measured using a stadiometer).

*Genotyping and instrument for genetically-determined BMI*

Genotyping was conducted using a custom-designed 800K-Single nucleotide polymorphism (SNP) array (Axiom [Affymetrix]), with imputation to 1000 Genomes Phase 3.^3^ Data were available for samples from ~100,000 participants aged 30-79 which had passed quality control (overall call rate >99.97% across all variants), including a population-based sample of 75,920 participants randomly selected from the total cohort (**Figure S1**).

A trans-ethnic BMI genetic score (BMI-GS) was developed using dosages of 851 bi-allelic SNPs with minor allele frequency ≥0.01 extracted from the sex-stratified genome-wide association study (GWAS) of BMI in CKB and UK Biobank (UKB).^4^ The BMI-GS (in standard deviation [SD] units) was a strong instrument (variance explained=5.21%) and was not associated with traits that might be considered potential confounders, including smoking and alcohol (**Table S2**). Separate sensitivity analyses used a BMI-GS developed using dosages of 789 bi-allelic SNPs with minor allele frequency ≥0.01 that were extracted from the sex-stratified GWAS of BMI in UKB (variance explained=2.30%).

*Follow-up for morbidity and mortality*

Information on incident disease events was obtained from linkage, via unique personal ID numbers, to electronic health insurance records, supplemented, for stroke, ischaemic heart disease (IHD), diabetes, and cancer, by linkage to disease registries. Mortality was monitored through China’s DSP system, with annual checks against local residential and administrative records. Underlying causes of death and disease were coded according to International Classification of diseases, 10^th^ revision (ICD-10) by trained staff blinded to baseline information.

For the present study, the main disease outcomes examined were incidence of major vascular events (MVE), IHD, ischaemic stroke (IS) and intracerebral haemorrhage (ICH), in addition to CVD, non-CVD and all-cause mortality (**Table S3**). Analyses were restricted to events occurring at ages 35-79 years, with censoring at the event of interest, 80 years of age or loss to follow-up.

*Statistical analyses*

*Conventional analyses*: Cox proportional hazards models, with time since recruitment as the timescale, were used to estimate hazard ratios (HRs) for disease outcomes, stratified by age-at-risk (5-year groups), sex, and study area (10 groups), and adjusted for education (no formal education, primary school, middle school, high school, college/university), smoking (never, occasional, ex-regular, current regular), alcohol intake (never, occasional intake, ex-regular, reduced intake, weekly intake) and physical activity (metabolic equivalent of task [MET] hours per day).^5^ The floating absolute risk method was used to estimate group-specific 95% CIs for each log HR to enable comparisons between any two categories (rather than just pairwise comparisons with the reference category).^6^

# Table S1. Reported conventional and genetic associations of BMI with risks of different CVDs in major published studies and meta-analyses

| **Outcome** | **Author** | **Study** | **No. of participants** | **No. of events** | **HR (95% CI)**  **per SD** | **HR (95% CI)**  **per 5 kg/m^2^** |
| --- | --- | --- | --- | --- | --- | --- |
| **Conventional analyses** | |  |  |  |  |  |
| **IHD** | Dale^7^ | ERFC^a^ | 143,710 | 5259 | 1.29 (1.22-1.37) | 1.32 (1.24-1.40) |
|  |  |  |  |  |  |  |
| **IS** | Dale^7^ | ERFC^a^ | 85,169 | 2431 | 1.20 (1.12-1.28) | 1.22 (1.13-1.31) |
|  | Park^8^ | Korean women study | 439,582 | 181,637 | N/A | 1.28 (1.27-1.28) |
|  | Song^9^ | Korean men study^b^ | 234,863 | 8696 | 1.30 (1.28-1.33) | 1.69 (1.65-1.72) |
|  |  |  |  |  |  |  |
| **ICH** | Park^8^ | Korean women study | 439,582 | 3953 | N/A | 1.10 (1.09-1.12) |
|  | Song^9^ | Korean men study^b^ | 234,863 | 1806 | 1.19 (1.17-1.21) | 1.40 (1.38-1.43) |
| **Genetic analyses** | |  |  |  |  |  |
| **IHD** | Dale^7^ | CARDIoGRAMplusC4D | 228,952 | 66,842 | 1.36 (1.22-1.52) | 1.40 (1.24-1.57) |
|  |  |  |  |  |  |  |
|  |  |  |  |  |  |  |
| **IS** | Dale^7^ | CARDIoGRAMplusC4D | 74,393 | 12,389 | 1.09 (0.93-1.28) | 1.10 (0.92-1.31) |
|  | Harshfield^10^ | MEGASTROKE^a^ | 454,450 | 60,341 | 1.14 (1.06-1.22) | 1.15 (1.07-1.24) |
|  | Larsson^11^ | METASTROKE & SGN^a^ | 37,296 | 18,476 | 1.11 (0.98-1.27) | 1.12 (0.97-1.29) |
|  | Marini^12^ | UKB & GIANT, MEGASTROKE & ISGC^c^ | N/A | 10,307 | 0.99 (0.92-1.07) | N/A |
|  |  |  |  |  |  |  |
| **ICH** | Harshfield^10^ | MEGASTROKE | 454,450 | 1545 | 1.26 (0.90-1.75) | 1.29 (0.90-1.85) |
|  |  |  |  |  |  |  |
| **ICH non-lobar** | Marini^12^ | UKB & GIANT, MEGASTROKE & ISGC | N/A | N/A- | 1.03 (0.58-1.82) | N/A |
|  |  |  |  |  |  |  |
| **ICH lobar** | Marini^12^ | UKB & GIANT, MEGASTROKE & ISGC | N/A | N/A | 1.35 (0.76-2.38) | N/A |

Abbreviations: CARDIoGRAMplusC4D=Coronary Artery Disease Genome wide Replication and Meta-analysis plus The Coronary Artery Disease, BMI= body mass index, CI= confidence limits, ERFC=Emerging Risk Factors Collaboration, GIANT=Genetic Investigation of ANtropometric Traits, HR= hazard ratio, ICH= intracerebral haemorrhage, IHD= ischaemic heart disease, IS= ischaemic stroke, ISGC=International Stroke Genetics Consortium, N/A= not applicable, SD= standard deviation, UKB=UK Biobank

^a^ The results were reported as odds ratios (95% CIs) per SD (4.6 kg/m^2^);

^b^ BMI SD is 2.54 kg/m^2^;

^c^ The results were reported as odds ratios and 95% confidence intervals for each 6% increment in BMI, corresponding to one SD.

.

# Table S2. Baseline characteristics by tertiles of BMI genetic score in the random genotyping subset

| **Characteristics** |  | **BMI genetic score tertiles** | | |
| --- | --- | --- | --- | --- |
|  |  | **T1**  **(n= 25,327)** | **T2**  **(n=25,328)** | **T3**  **(n=25,327)** |
| Age, years (SD) |  | 52.2 (10.6) | 52.3 (10.6) | 52.2 (10.5) |
| Women, % |  | 59.6 | 59.9 | 59.9 |
| Urban, % |  | 48.3 | 48.3 | 48.3 |
| ≥6 years of education, % |  | 51.3 | 50.7 | 50.8 |
| Ever regular smoker, % |  |  |  |  |
| Men |  | 73.1 | 74.1 | 74.1 |
| Women |  | 2.9 | 3.3 | 3.7 |
| Regular alcohol consumption**, %** |  |  |  |  |
| Men |  | 37.9 | 39.1 | 38.7 |
| Women |  | 2.7 | 2.6 | 2.5 |
| Physical activity, MET-h/day (SD) |  | 20.6 (11.5) | 20.5 (11.8) | 20.4 (11.8) |

Adjusted for age, sex and study area, as appropriate. BMI=body mass index, MET=metabolic equivalent of task.

BMI= body mass index, MET= metabolic equivalent of task, SD; standard deviation

# Table S3. ICD-10 codes for disease outcomes analysed

| Outcomes | ICD-10 codes |
| --- | --- |
| CVD mortality | I00-I99 |
| Non-CVD mortality | A00-B99, C00-D49, D50-D89, E00-E89, F01-F99, G00-G99, H00-H59, H60-H95, J00-J99, K00-K95, L00-L99, M00-M99, N00-N99, O00-O9A, Q00-Q99, R00-R99, S00-T88 |
| All-cause mortality | A00-B99, C00-D49, D50-D89, E00-E89, F01-F99, G00-G99, H00-H59, H60-H95, J00-J99, I00-I99, K00-K95, L00-L99, M00-M99, N00-N99, O00-O9A, Q00-Q99, R00-R99, S00-T88 |
| Major vascular events incidence | I21-I23, I60-I64, and Fatal I00-I20, I24-I52, I65-I68, I70-I78 |
| Ischaemic heart disease | I20-I25 |
| Ischaemic stroke | I63 |
| Intracerebral haemorrhage | I61 |
| Non-CVD mortality is any underlying cause of death apart from CVD death.  CVD=cardiovascular disease | |

# Table S4. Characteristics of participants by measured BMI at baseline in the random genotyping subset

| **Characteristics** | **BMI, kg/m^2^** | | | | | | | | **All**  (n=75,981) |
| --- | --- | --- | --- | --- | --- | --- | --- | --- | --- |
|  | **<20**  (n=9578) | | | **20.0 to <25.0**  (n=40,423) | **25.0 to <30.0**  (n=22,469) | | **≥30.0**  (n=3511) | |  |
| **Age and socioeconomic factors** |  | |  |  | |  |  |  |  |
| Age, years (SD) | 52.7 (13.8) | | | 51.6 (10.6) | 52.3 (10.4) | | 52.3 (12.9) | | 52.2 (10.6) |
| Women, % | 56.2 | | | 59.1 | 60.9 | | 70.3 | | 59.8 |
| Urban, % | 34.2 | | | 45.0 | 57.6 | | 65.8 | | 48.3 |
| ≥6 years of education, % | 50.4 | | | 51.3 | 51.5 | | 49.6 | | 51.0 |
| **Anthropometry, blood pressure and heart rate, mean (SD)** | | | | |  | |  | |  |
| BMI, kg/m^2^ | 18.8 (1.3) | | | 22.6 (1.4) | 26.8 (1.4) | | 31.8 (2.3) | | 23.6 (3.2) |
| Waist circumference, cm | 68.3 (5.9) | | | 77.7 (5.9) | 88.1 (6.2) | | 99.0 (8.9) | | 80.3 (9.0) |
| Waist-to-hip ratio | 0.82 (0.07) | | | 0.87 (0.06) | 0.92 (0.06) | | 0.95 (0.07) | | 0.88 (0.06) |
| Percentage body fat | 18.9 (3.7) | | | 26.2 (4.0) | 33.5 (4.6) | | 40.1 (6.9) | | 27.9 (6.4) |
| Systolic blood pressure, mmHg | 123 (24) | | | 129 (19) | 136 (21) | | 143 (28) | | 131 (20) |
| Heart rate, bpm | 79 (15) | | | 78 (12) | 80 (12) | | 82 (17) | | 79 (11) |
| **Lifestyle factors** | |  | |  |  | |  | |  |
| Ever regular smoker**, %** | |  | |  |  | |  | |  |
| Men | | 79.4 | | 74.3 | 70.2 | | 70.1 | | 73.8 |
| Women | | 4.7 | | 3.3 | 3.0 | | 3.3 | | 3.3 |
| Regular alcohol consumption**, %** | |  | |  |  | |  | |  |
| Men | | 38.0 | | 38.8 | 38.7 | | 39.1 | | 38.6 |
| Women | | 2.5 | | 2.7 | 2.7 | | 1.8 | | 2.6 |
| Physical activity, MET-h/day (SD) | | 20.8 (14.0) | | 20.9 (11.6) | 19.9 (12.4) | | 18.1 (15.7) | | 20.5 (11.7) |
| **Medical history, %** | | | |  |  | |  | |  |
| Diabetes^a^ | | 3.4 | | 5.3 | 8.0 | | 11.3 | | 6.2 |
| Cardiovascular diseases | | 2.8 | | 3.9 | 5.6 | | 7.1 | | 4.1 |
| Uncontrolled hypertension^b^ | | 4.4 | | 9.2 | 17.3 | | 25.9 | | 11.8 |
| Chronic respiratory disease | | 15.3 | | 8.8 | 6.8 | | 6.8 | | 9.1 |
| Chronic liver disease | | 1.6 | | 1.3 | 1.1 | | 0.8 | | 1.3 |
| Chronic kidney disease | | 1.4 | | 1.5 | 1.7 | | 1.4 | | 1.5 |
| Cancer^c^ | | 0.3 | | 0.1 | 0.1 | | 0.2 | | 0.2 |
| Self-rated poor health | | 14.1 | | 9.4 | 9.8 | | 13.6 | | 10.3 |
| Adjusted for age, sex and study area, as appropriate. BMI= body mass index, DBP= diastolic blood pressure, MET= metabolic equivalent of task, SBP= systolic blood pressure, SD= standard deviation. ^a^Self-reported or screen-detected diabetes; ^b^Self-reported hypertension and SBP ≥140 mmHg or DBP ≥90mmHg; ^c^Self-reported cancer and on treatment at baseline. | | | | | | | | | |

# Table S5. Conventional associations of BMI with risks of CVD incidence and CVD, non-CVD and all-cause mortality

| **BMI strata** | **Mean**  **BMI, kg/m^2^** | | **No. of**  **events** | **HR (95% CI)^a^** |
| --- | --- | --- | --- | --- |
| **Incidence** |  |  | |  |
| **Major vascular events** |  |  | |  |
| <20.0 | 18.7 | 9179 | | 0.95 (0.93-0.97) |
| 20.0-24.9 | 22.6 | 33610 | | 1.00 (0.99-1.01) |
| 25.0-29.9 | 26.9 | 22010 | | 1.24 (1.23-1.26) |
| ≥30.0 | 31.9 | 3632 | | 1.47 (1.43-1.52) |
| *Test for non-linearity across BMI range* |  |  | | *X^2^=79.94, P<0.0001* |
| *Test for non-linearity at BMI ≥20 kg/m^2^* |  |  | | *X^2^=78.03, P=0.01* |
| **Ischaemic heart disease** |  |  | |  |
| <20.0 | 18.7 | 5996 | | 0.93 (0.90-0.95) |
| 20.0-24.9 | 22.6 | 23872 | | 1.00 (0.99-1.01) |
| 25.0-29.9 | 26.9 | 17525 | | 1.31 (1.29-1.33) |
| ≥30.0 | 31.9 | 3305 | | 1.66 (1.61-1.72) |
| *Test for non-linearity across BMI range* |  |  | | *X^2^=78.03, P<0.0001* |
| *Test for non-linearity at BMI ≥20 kg/m^2^* |  |  | | *X^2^=13.10, P=0.01* |
| **Ischaemic stroke** |  |  | |  |
| <20.0 | 18.7 | 4256 | | 0.77 (0.75-0.80) |
| 20.0-24.9 | 22.6 | 20938 | | 1.00 (0.99-1.01) |
| 25.0-29.9 | 26.9 | 14763 | | 1.27 (1.25-1.30) |
| ≥30.0 | 31.9 | 2470 | | 1.50 (1.45-1.57) |
| *Test for non-linearity across BMI range* |  |  | | *X^2^=95.33, P<0.0001* |
| *Test for non-linearity at BMI ≥20 kg/m^2^* |  |  | | *X^2^=12.38, P=0.01* |
| **Intracerebral haemorrhage** |  |  | |  |
| <20.0 | 18.7 | 1330 | | 0.99 (0.94-1.05) |
| 20.0-24.9 | 22.6 | 3943 | | 1.00 (0.97-1.03) |
| 25.0-29.9 | 26.9 | 2065 | | 1.20 (1.14-1.25) |
| ≥30.0 | 31.9 | 306 | | 1.43 (1.28-1.60) |
| *Test for non-linearity across BMI range* |  |  | | *X^2^=91.67, P<0.0001* |
| *Test for non-linearity at BMI ≥20 kg/m^2^* |  |  | | *X^2^=12.15, P=0.01* |
|  |  |  | |  |
| **Mortality** |  |  | |  |
| **CVD** |  |  | |  |
| <20.0 | 18.7 | 2949 | | 1.10 (1.06-1.14) |
| 20.0-24.9 | 22.6 | 7672 | | 1.00 (0.98-1.02) |
| 25.0-29.9 | 26.9 | 4092 | | 1.13 (1.09-1.17) |
| ≥30.0 | 31.9 | 714 | | 1.48 (1.37-1.59) |
| *Test for non-linearity across BMI range range* |  |  | | *X^2^=61.28, P<0.0001* |
| *Test for non-linearity at BMI ≥20 kg/m^2^* |  |  | | *X^2^=5.48, P=0.02* |
| **Non-CVD** |  |  | |  |
| <20.0 | 18.7 | 6158 | | 1.50 (1.46-1.54) |
| 20.0-24.9 | 22.6 | 13771 | | 1.00 (0.98-1.02) |
| 25.0-29.9 | 26.9 | 6087 | | 0.88 (0.86-0.91) |
| ≥30.0 | 31.9 | 899 | | 0.99 (0.93-1.06) |
| *Test for non-linearity across BMI range* |  |  | | *X^2^=295.82, P<0.0001* |
| *Test for non-linearity at BMI ≥20 kg/m^2^* |  |  | | *X^2^=30.12, p<0.0001* |
| **All-cause** |  |  | |  |
| <20.0 | 18.7 | 9107 | | 1.34 (1.31-1.37) |
| 20.0-24.9 | 22.6 | 21443 | | 1.00 (0.99-1.01) |
| 25.0-29.9 | 26.9 | 10179 | | 0.97 (0.95-0.99) |
| ≥30.0 | 31.9 | 1613 | | 1.16 (1.11-1.22) |
| *Test for non-linearity across BMI range* |  |  | | *X^2^=368.23, P<0.0001* |
| *Test for non-linearity at BMI ≥20 kg/m^2^* |  |  | | *X^2^=37.36, P<0.0001* |

^a^ Cox proportional hazard models were used (stratified by age-at-risk, sex and study area, and adjusted for smoking, alcohol, education and physical activity) to estimate the conventional association of baseline BMI with outcomes.

BMI= body mass index, CVD= cardiovascular disease, HR= hazard ratio, CI= confidenceintervals

# Table S6. Genetic associations of BMI with risks of CVD, non-CVD and all-cause mortality

| **BMI strata^a^** | **BMI, kg/m^2^** | | **GS-BMI^b^**  **Estimate (SE)** | **No. of deaths** | **GS-Outcome^c^**  **Estimate (SE)** | **LACE^d^**  **Estimate (SE)** |
| --- | --- | --- | --- | --- | --- | --- |
|  | **Range** | **Mean** |  |  |  |  |
| **CVD mortality** |  |  |  |  |  |  |
| Strata 1 | 12.6-26.3 | 18.7 | 2.07 (0.064) | 1081 | 0.09 (0.113) | 0.045 (0.055) |
| Strata 2 | 15.2-27.0 | 20.6 | 2.31 (0.056) | 823 | 0.32 (0.129) | 0.138 (0.056) |
| Strata 3 | 17.1-27.4 | 21.5 | 2.49 (0.053) | 833 | 0.09 (0.127) | 0.036 (0.051) |
| Strata 4 | 17.7-28.3 | 22.4 | 2.70 (0.052) | 739 | 0.27 (0.135) | 0.101 (0.050) |
| Strata 5 | 18.9-29.0 | 23.2 | 2.81 (0.053) | 718 | 0.29 (0.138) | 0.104 (0.049) |
| Strata 6 | 24.0-19.2 | 24.0 | 2.90 (0.055) | 649 | 0.55 (0.146) | 0.189 (0.050) |
| Strata 7 | 24.9-19.7 | 24.9 | 3.06 (0.058) | 685 | 0.47 (0.142) | 0.153 (0.046) |
| Strata 8 | 25.9-20.7 | 25.9 | 3.25 (0.063) | 655 | 0.28 (0.143) | 0.086 (0.044) |
| Strata 9 | 27.2-21.5 | 27.2 | 3.34 (0.074) | 711 | 0.26 (0.142) | 0.077 (0.042) |
| Strata 10 | 22.4-47.1 | 31.5 | 3.72 (0.107) | 740 | 0.42 (0.137) | 0.112 (0.037) |
| *Test for heterogeneity* |  |  | *X^2^=447.6, P<0.001* |  | *X^2^=11.1, P=0.266* | *X^2^=7.8, P=0.551* |
| **Non-CVD mortality** |  |  |  |  |  |  |
| Strata 1 | 12.6-26.3 | 18.7 | 2.07 (0.064) | 758 | 0.027 (0.137) | 0.131 (0.066) |
| Strata 2 | 15.2-27.0 | 20.6 | 2.31 (0.056) | 528 | -0.21 (0.160) | -0.089 (0.069) |
| Strata 3 | 17.1-27.4 | 21.5 | 2.49 (0.053) | 440 | 0.15 (0.179) | 0.062 (0.072) |
| Strata 4 | 17.7-28.3 | 22.4 | 2.70 (0.052) | 420 | 0.25 (0.181) | 0.094 (0.067) |
| Strata 5 | 18.9-29.0 | 23.2 | 2.81 (0.053) | 401 | 0.32 (0.184) | 0.115 (0.065) |
| Strata 6 | 24.0-19.2 | 24.0 | 2.90 (0.055) | 359 | 0.19 (0.199) | 0.065 (0.069) |
| Strata 7 | 24.9-19.7 | 24.9 | 3.06 (0.058) | 356 | 0.01 (0.199) | 0.002 (0.065) |
| Strata 8 | 25.9-20.7 | 25.9 | 3.25 (0.063) | 351 | 0.13 (0.200) | 0.041 (0.062) |
| Strata 9 | 27.2-21.5 | 27.2 | 3.34 (0.074) | 360 | 0.24 (0.201) | 0.072 (0.060) |
| Strata 10 | 22.4-47.1 | 31.5 | 3.72 (0.107) | 382 | 0.46 (0.193) | 0.124 (0.052) |
| *Test for heterogeneity* |  |  | *X^2^=447.6, P<0.001* |  | *X^2^=10.1, P=0.0348* | *X^2^=9.1, P=0.431* |
| **All-cause mortality** |  |  |  |  |  |  |
| Strata 1 | 12.6-26.3 | 18.7 | 2.07 (0.064) | 1059 | 0.23 (0.115) | 0.111 (0.056) |
| Strata 2 | 15.2-27.0 | 20.6 | 2.31 (0.056) | 772 | -0.01 (0.133) | -0.006 (0.058) |
| Strata 3 | 17.1-27.4 | 21.5 | 2.49 (0.053) | 658 | 0.16 (0.145) | 0.065 (0.058) |
| Strata 4 | 17.7-28.3 | 22.4 | 2.70 (0.052) | 636 | 0.25 (0.146) | 0.093 (0.054) |
| Strata 5 | 18.9-29.0 | 23.2 | 2.81 (0.053) | 616 | 0.43 (0.149) | 0.154 (0.053) |
| Strata 6 | 24.0-19.2 | 24.0 | 2.90 (0.055) | 584 | 0.21 (0.156) | 0.073 (0.054) |
| Strata 7 | 24.9-19.7 | 24.9 | 3.06 (0.058) | 603 | 0.18 (0.152) | 0.058 (0.049) |
| Strata 8 | 25.9-20.7 | 25.9 | 3.25 (0.063) | 572 | 0.24 (0.157) | 0.073 (0.048) |
| Strata 9 | 27.2-21.5 | 27.2 | 3.34 (0.074) | 598 | 0.20 (0.156) | 0.060 (0.047) |
| Strata 10 | 22.4-47.1 | 31.5 | 3.72 (0.107) | 686 | 0.56 (0.144) | 0.151 (0.039) |
| *Test for heterogeneity* |  |  | *X^2^=447.6, P<0.001* |  | *X^2^=10.9, P=0.286* | *X^2^=8.1, P=0.526* |

^a^BMI strata refer to doubly-ranked strata.

^b^The estimates of BMI in kg/m^2^ per 1 GS SD (from the association of BMI GS and BMI) across the BMI strata, in the random genotyping subset.

^c^The log HR per 1 GS SD from Cox proportional hazard models (stratified by age-at-risk, sex and study area) to estimate the association of BMI GS with outcomes across BMI strata. This analysis was conducted among the random genotyping subset and CVD cases combined.

^d^The LACE estimates (log HR per average GS 1 kg/m^2^) are calculated as the ratio of the log HR from the GS-outcome association and the estimate from the BMI GS association, across BMI strata.

BMI= body mass index, CVD= cardiovascular disease, GS= genetic score, HR= hazard ratio, LACE= localised average causal effect, SE= standard error, SD= standard deviation

# Figure S1. Flow diagram of participants included in (A) conventional and (B) genetic analyses

BMI= body mass index, CVD= cardiovascular disease

1. **Conventional analyses**
2. **Genetic analyses**

Participants with CVD cases selected for genotyping (n=12,809)

Participants in the random genotyping subset (n=75,982)

Excluded participants with missing BMI (n=1)

All participants (n=512,724)

Excluded participants with missing BMI (n=2)

Excluded participants prior self-reported doctor diagnosed CVD (n=23,129)

Participants in genetic analyses for non-CVD and

all-cause mortality (n=75,981)

Participants in conventional analyses (n=489,593)

Participants in genetic analyses for CVD outcomes (n=88,790)

# Figure S2. (A) Conventional and (B) genetic associations of BMI with risks of non-CVD and all-cause mortality

The HRs in conventional analyses were stratified by age-at-risk (5-year age groups), sex, and study area (ten groups), and adjusted for education (5 groups), smoking (4 groups), alcohol intake (5 groups) and physical activity (metabolic equivalent of task [MET] hours per day) and are shown as solid squares and 95% CI as vertical lines. In MR analyses, the fractional polynomial method was used to estimate relationship between BMI and outcomes (stratified by age-at-risk [5-year age groups], sex, and study area [10 groups], and adjusted for 11 genetic PCs). The reference was set to mean BMI (23.8 kg/m^2^). The 95% CI in genetic analyses are represented by the shaded patterns. BMI= body mass index, CI= confidence limits, CVD= cardiovascular disease, HR= hazard ratio


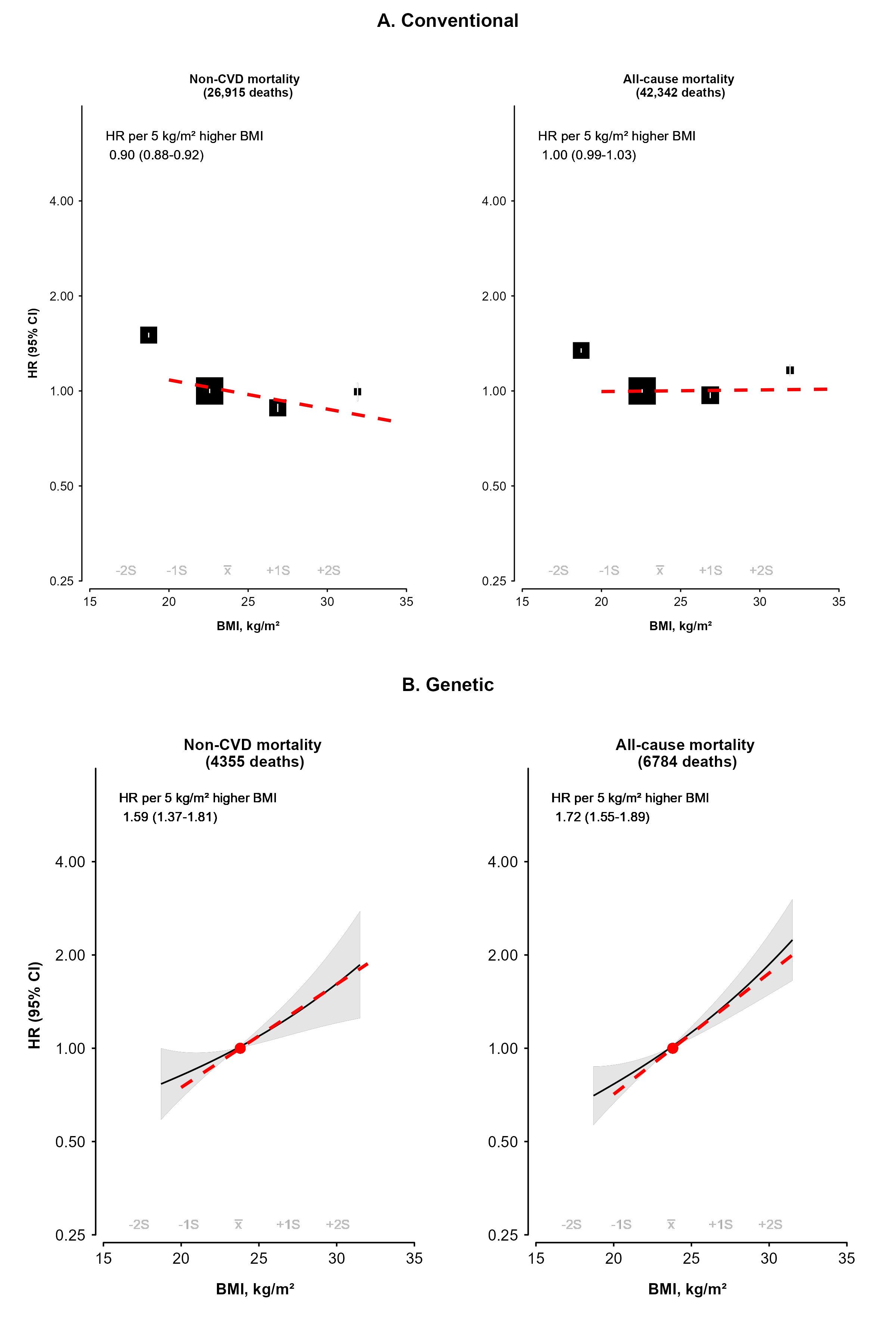


# Figure S3. Conventional associations of BMI with risks of CVD, non-CVD and all-cause mortality in (A) healthy and (B) unhealthy participants

The HRs for BMI were stratified by age-at-risk (5-year groups), sex, and study area (ten groups), and adjusted for education (5 groups), smoking (4 groups), alcohol intake (5 groups) and physical activity (MET-hours per day) and are shown as squares with 95% CI indicated by vertical lines. Healthy individuals were those without self-reported major chronic diseases (diabetes, CVD, chronic respiratory disease, chronic kidney disease, chronic liver disease, caner) or uncontrolled hypertension (self-reported and SBP ≥140 mmHg or DBP ≥90 mmHg) or poor self-rated health at recruitment. BMI= body mass index, CI= confidence limits, CVD= cardiovascular disease, HR= hazard ratio, MVE= major vascular events


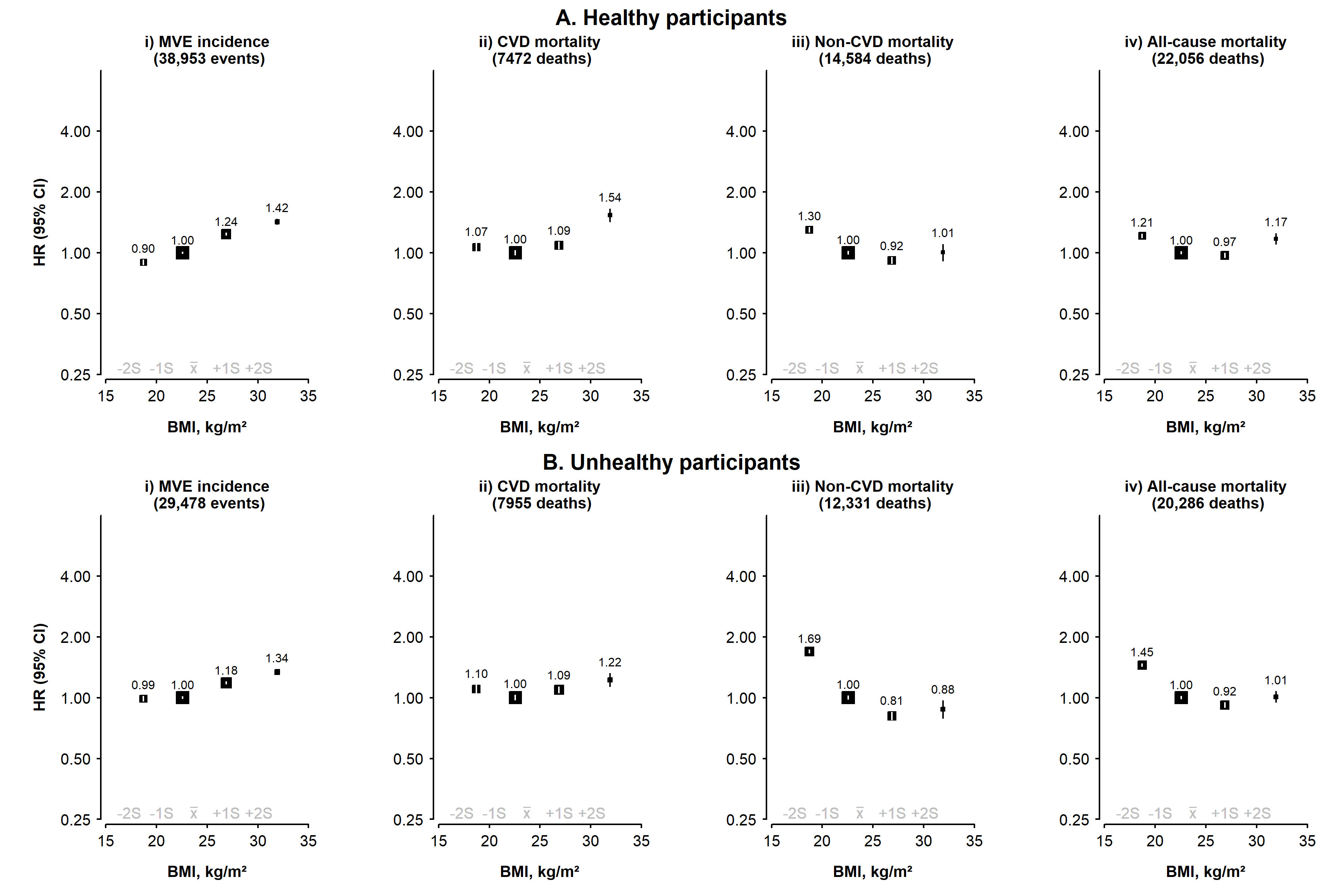


#

# Figure S4. Conventional associations of BMI with risks of CVD, non-CVD and all-cause mortality among healthy participants by smoking status

Conventions as in Figure S 3.


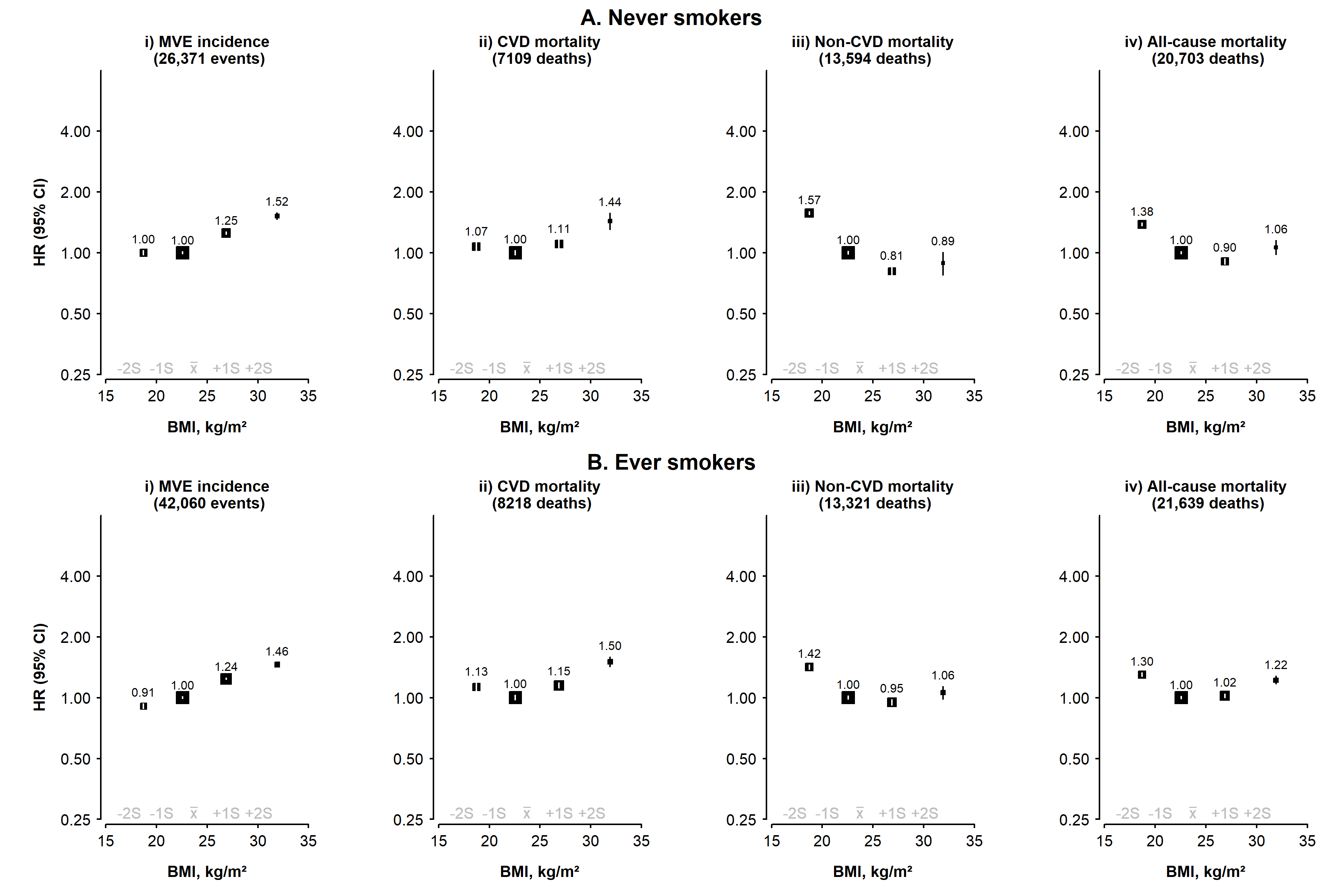


# Figure S5. Conventional associations of BMI with risks of CVD, non-CVD and all-cause mortality among healthy participants by years of follow-up

Conventions as in Figure S 3.


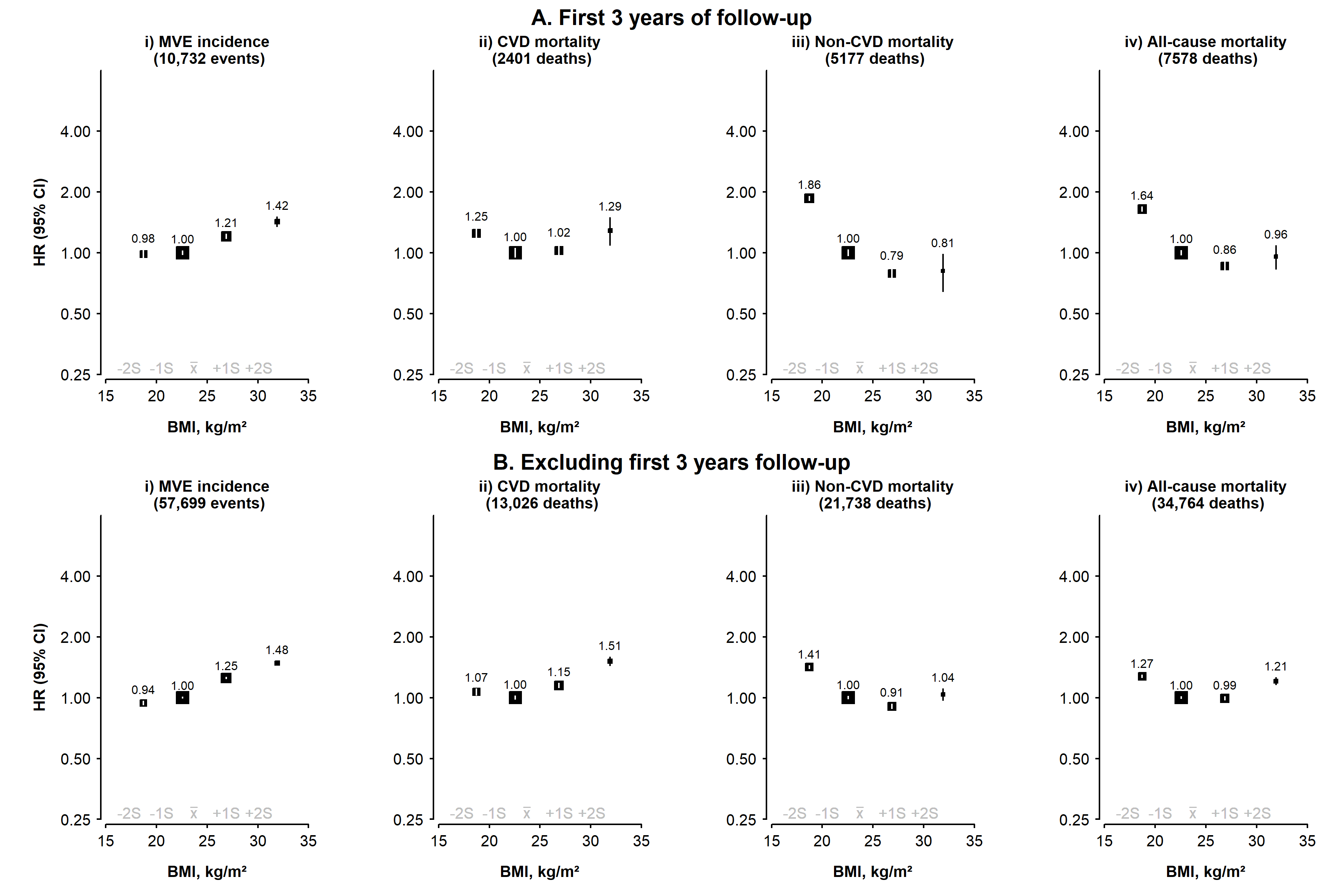


# Figure S6. Adjusted HRs for CVD and non-CVD mortality risks per 5 kg/m^2^ higher BMI at BMI ≥20.0 kg/m^2^ by age and sex in (A) conventional and (B) genetic analyses

The HRs are shown as squares and 95% CI as horizontal lines. The chi-square and P-values are shown for heterogeneity or linear trend between sex and age-specific groups, respectively. Conventional HRs for BMI were stratified by age-at-risk (5-year groups), sex, and study area (ten groups), and adjusted for education (5 groups), smoking (4 groups), alcohol intake (5 groups) and physical activity (MET-hours per day). Genetic HRs were estimates using the ratio method and stratified by age-at-risk (5-year groups), sex, and study area (ten groups), and adjusted for 11 genetic PCs. BMI= body mass index, CI= confidence limits, CVD= cardiovascular disease, HR= hazard ratio, ICH= intracerebral haemorrhage, IHD= ischaemic heart disease, IS= ischaemic stroke


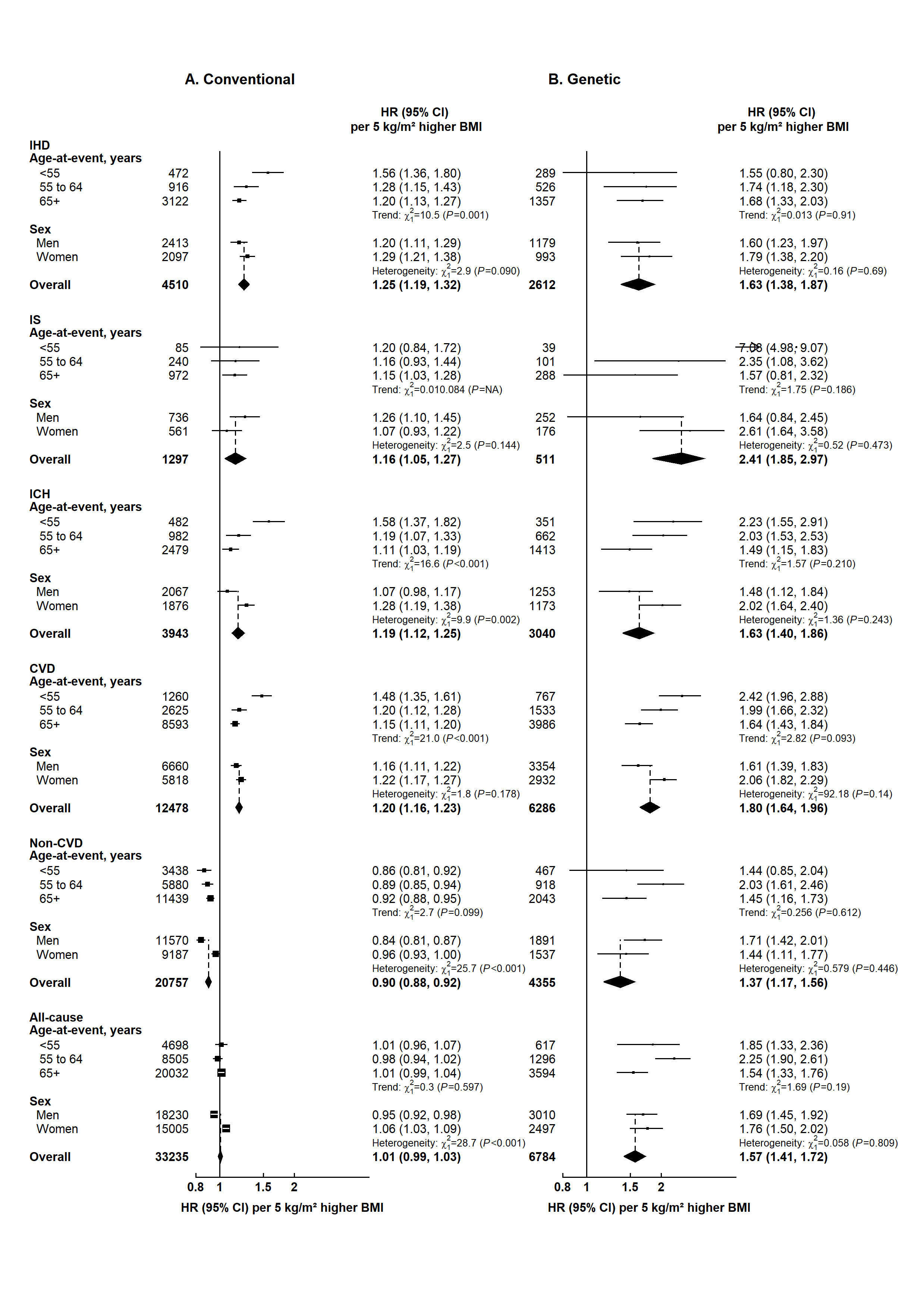


# Figure S7. Conventional associations of BMI with risks of CVD (A) incidence and (B) mortality using seven BMI categories

The HRs for BMI were stratified by age-at-risk (5-year groups), sex, and study area (ten groups), and adjusted for education (5 groups), smoking (4 groups), alcohol intake (5 groups) and physical activity (MET-hours per day) and are shown as solid squares and 95% CI as vertical lines. BMI= body mass index, CI= confidence limits, CVD= cardiovascular disease, HR= hazard ratio, ICH= intracerebral haemorrhage, IHD= ischaemic heart disease, IS= ischaemic stroke, MVE= major vascular disease


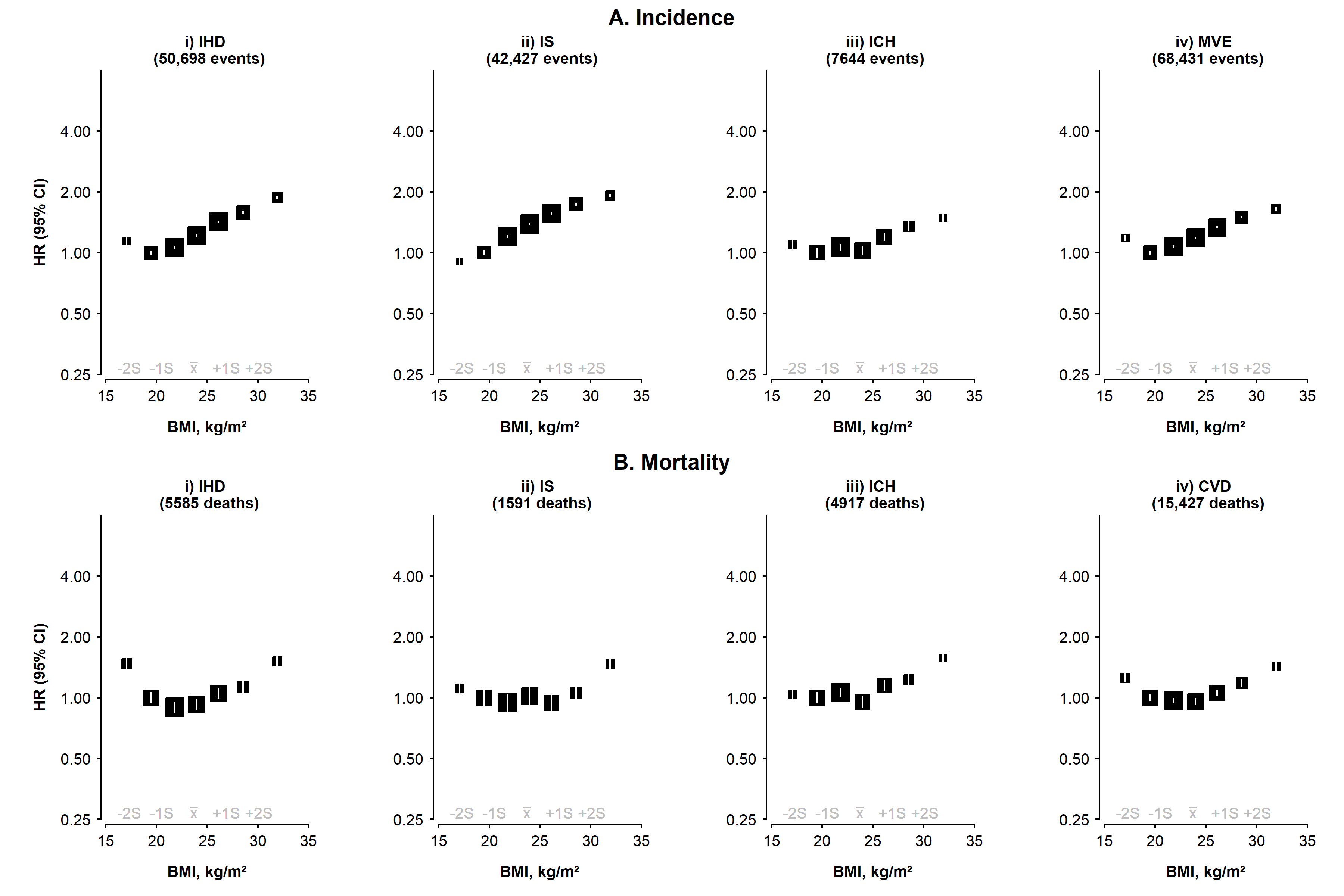


# Figure S8. Genetic associations of BMI with risks of CVD (A) incidence and (B) mortality excluding participants with self-reported doctor diagnosed CVD at baseline

Fractional polynomial method was used to estimate relationship between BMI and outcomes (stratified by age-at-risk [5-year age groups], sex, and study area [10 groups], and adjusted for 11 genetic PCs). The reference was set to mean BMI (23.8 kg/m^2^). The 95% CI in genetic analyses are represented by the shaded patterns. BMI= body mass index, CI= confidence limits, CVD= cardiovascular disease, HR= hazard ratio, ICH= intracerebral haemorrhage, IHD= ischaemic heart disease, IS= ischaemic stroke, MVE= major vascular disease


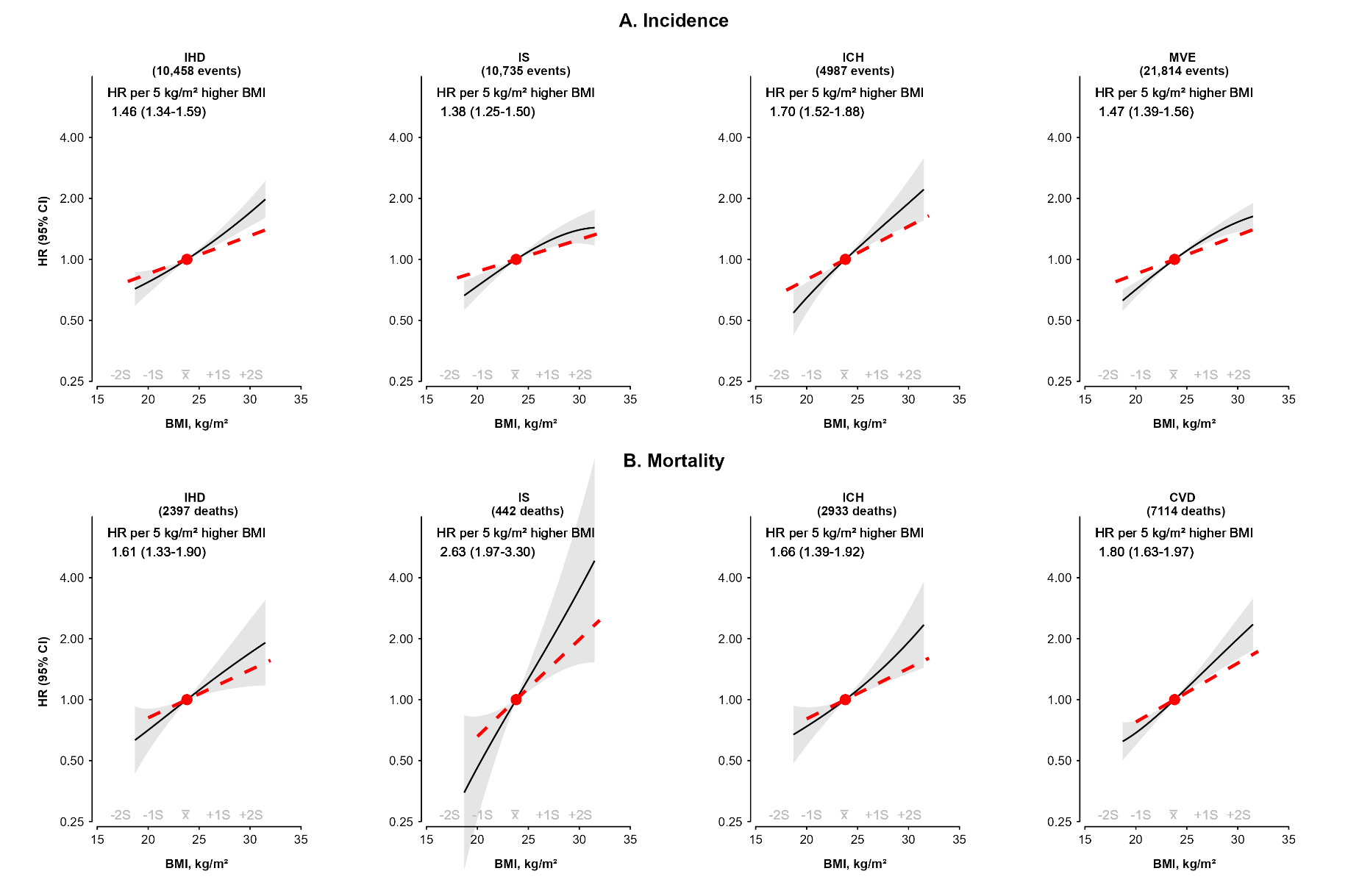


# Figure S9. Genetic associations of BMI with risks of CVD (A) incidence and (B) mortality estimating the denominator in the random genotyping subset only and in the random genotyping subset plus CVD cases

Fractional polynomial method was used to estimate relationship between BMI and outcomes (stratified by age-at-risk [5-year age groups], sex, and study area [10 groups], and adjusted for 11 genetic PCs). The reference was set to mean BMI (23.8 kg/m^2^). The 95% CI in genetic analyses are represented by the shaded patterns. BMI= body mass index, CI= confidence limits, CVD= cardiovascular disease, HR= hazard ratio, ICH= intracerebral haemorrhage, IHD= ischaemic heart disease, IS= ischaemic stroke, MVE= major vascular disease


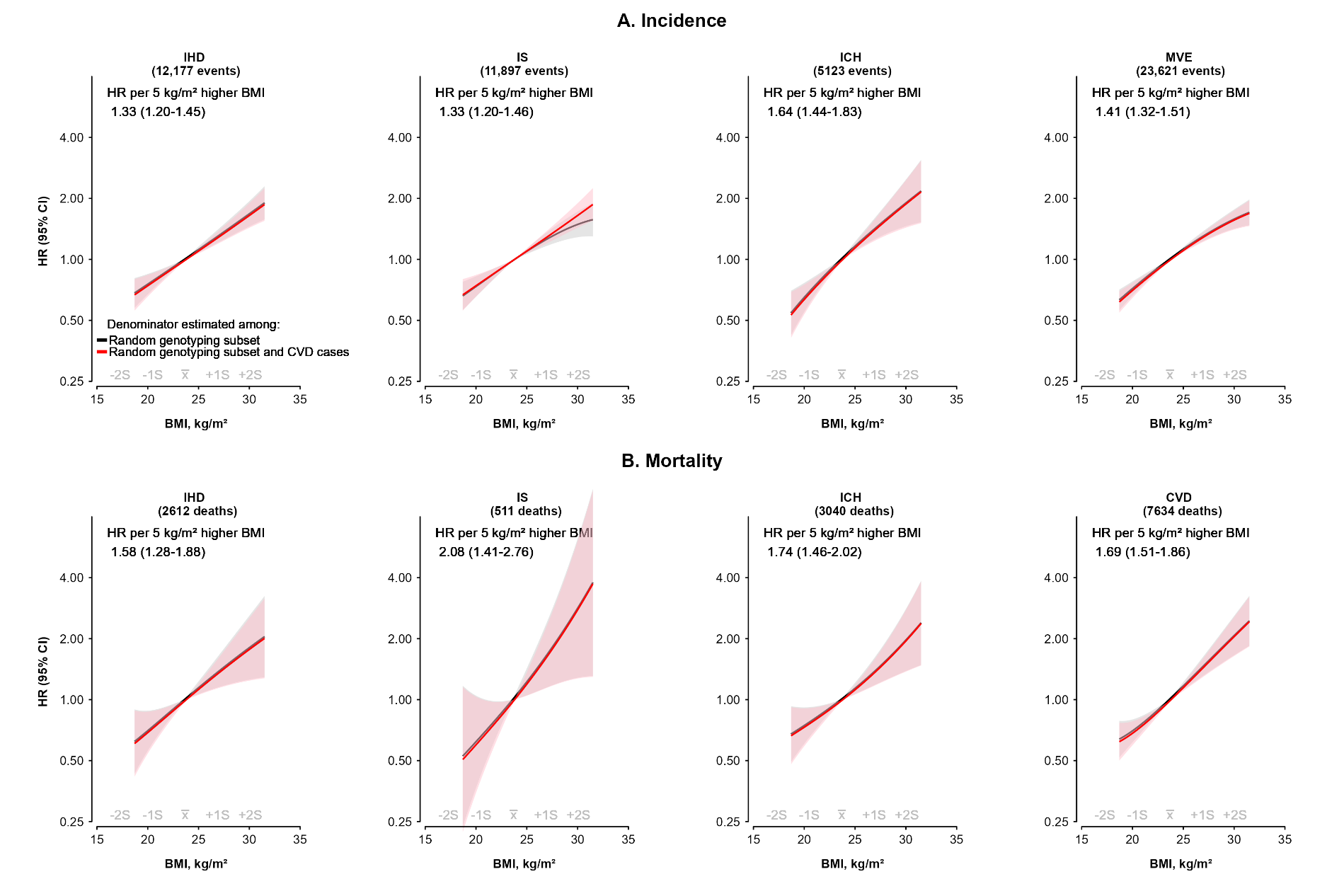


# Figure S10. Genetic associations of BMI with risks of CVD (A) incidence and (B) mortality additionally adjust for the variables included in the conventional analyses

Fractional polynomial method was used to estimate relationship between BMI and outcomes (stratified by age-at-risk [5-year age groups], sex, and study area [10 groups], and adjusted for education (no formal education, primary school, middle school, high school, college/university), smoking (never, occasional, ex-regular, current regular), alcohol intake (never, occasional intake, ex-regular, reduced intake, weekly intake) and physical activity (metabolic equivalent of task [MET] hours per day) and11 genetic PCs). The reference was set to mean BMI (23.8 kg/m^2^). The 95% CI in genetic analyses are represented by the shaded patterns. BMI= body mass index, CI= confidence limits, CVD= cardiovascular disease, HR= hazard ratio, ICH= intracerebral haemorrhage, IHD= ischaemic heart disease, IS= ischaemic stroke, MVE= major vascular disease


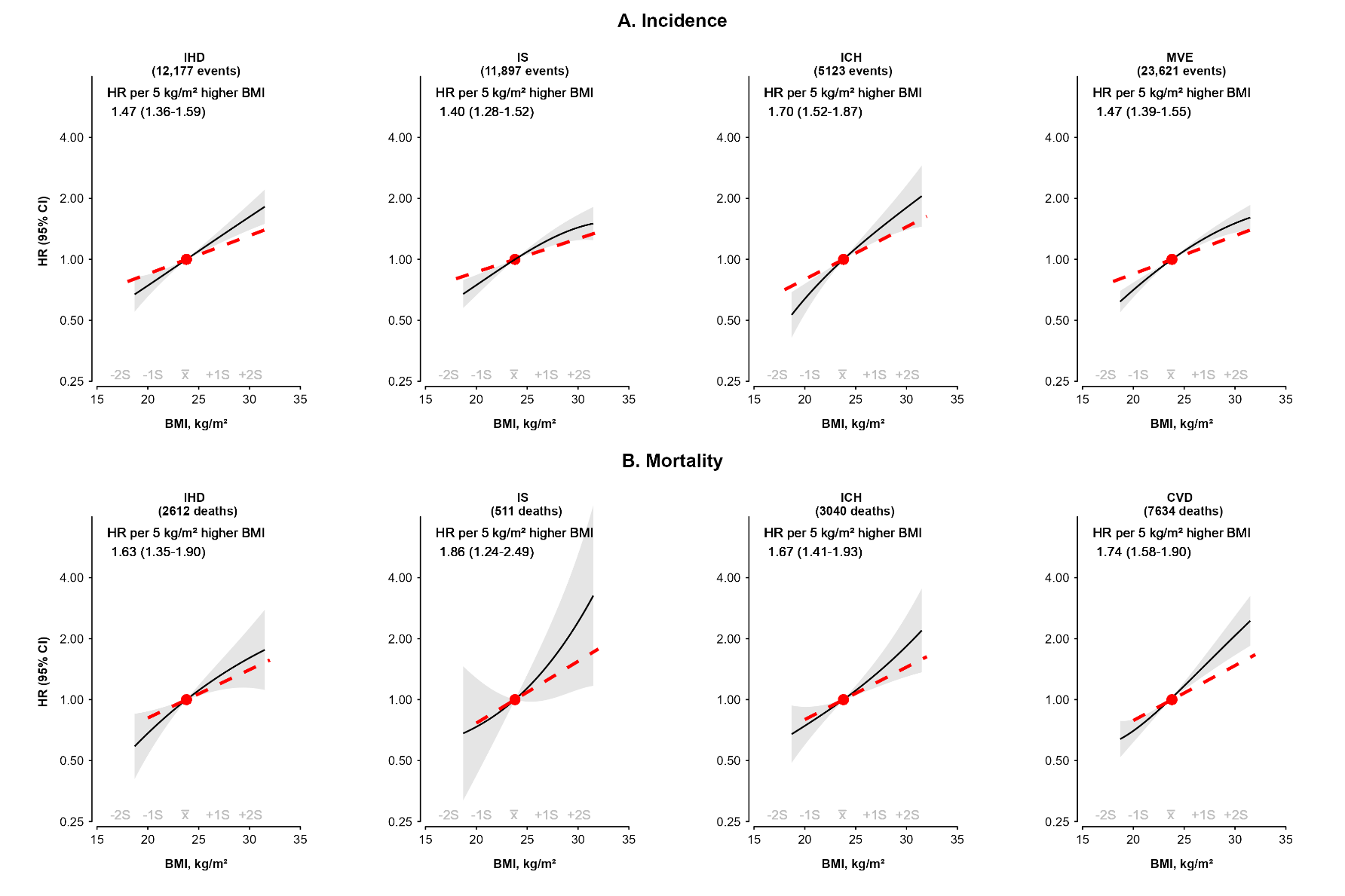


# Figure S11. Genetic associations of BMI with risks of CVD (A) incidence and (B) mortality using the doubly ranked method vs. the residual method

Fractional polynomial method was used to estimate relationship between BMI and outcomes (stratified by age-at-risk [5-year age groups], sex, and study area [10 groups], and adjusted for 11 genetic PCs). The reference was set to mean BMI (23.8 kg/m^2^). The 95% CI in genetic analyses are represented by the shaded patterns. BMI= body mass index, CI= confidence limits, CVD= cardiovascular disease, HR= hazard ratio, ICH= intracerebral haemorrhage, IHD= ischaemic heart disease, IS= ischaemic stroke, MVE= major vascular disease


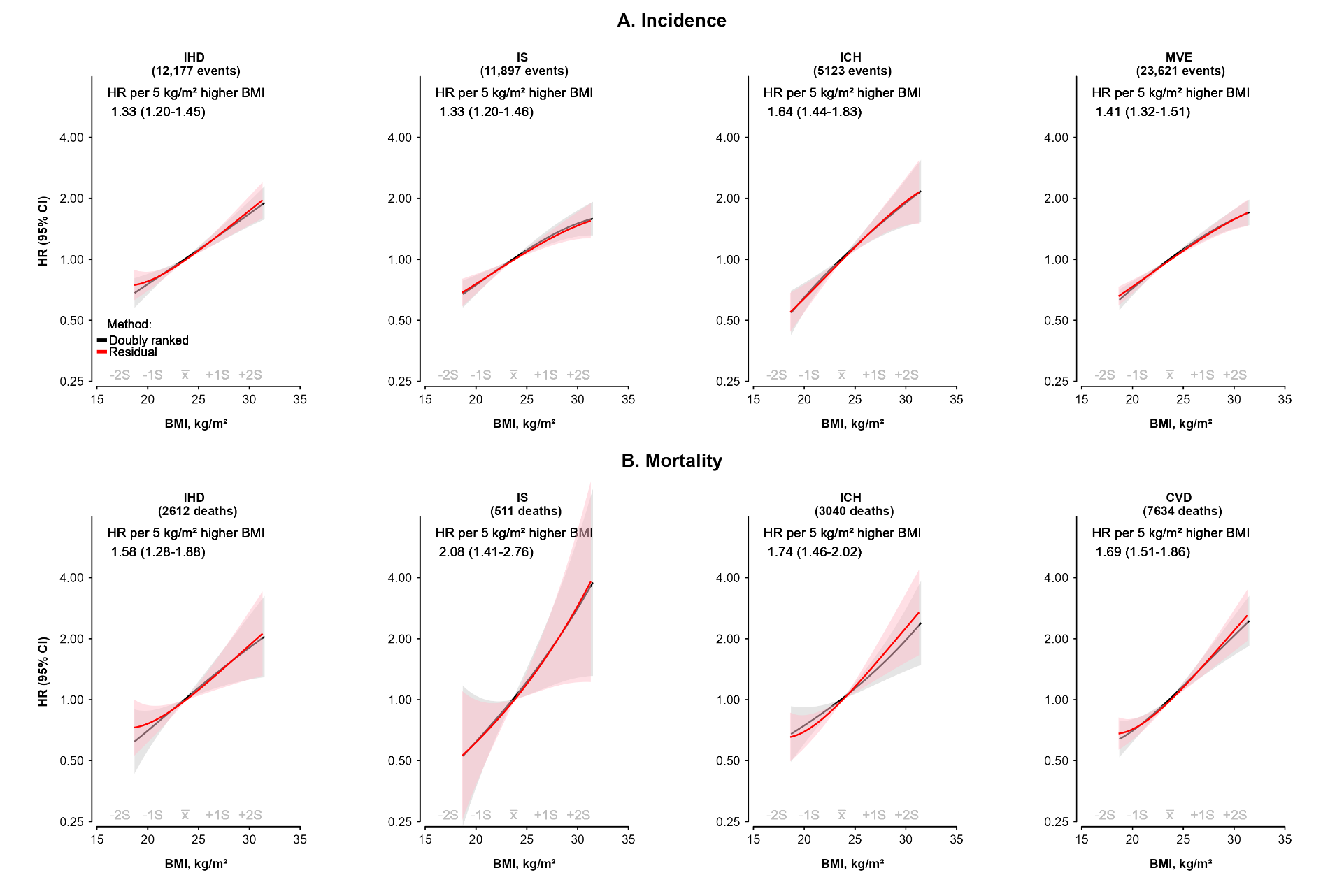


# Figure S12. Genetic associations of BMI with age and sex doubly ranked vs. residual method

Linear regression was used to estimate relationship between BMI GS and age and logistic regression to estimate relationship between BMI GS and sex. The reference was set to mean BMI (23.8 kg/m^2^). The 95% CI in genetic analyses are represented by the shaded patterns.

*Adjusted for age and sex where appropriate, study area and 11 genetic PCs. BMI= body mass index, GS= genetic score


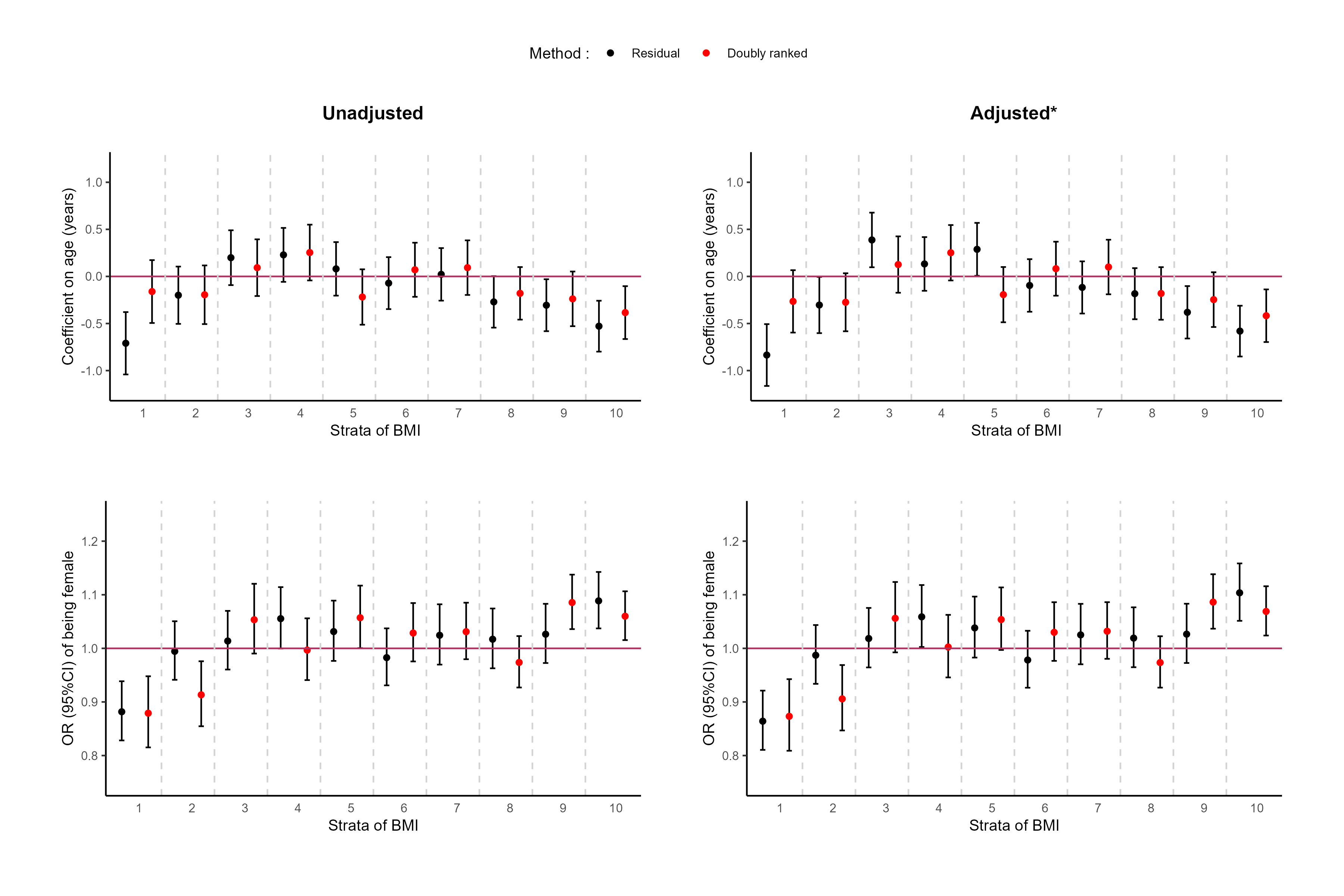


# Figure S13. Genetic associations of BMI with risks of CVD (A) incidence and (B) mortality using BMI genetic score with UKB SNPs and weights

Fractional polynomial method was used to estimate relationship between BMI and outcomes (stratified by age-at-risk [5-year age groups], sex, and study area [10 groups], and adjusted for 11 genetic PCs). The reference was set to mean BMI (23.8 kg/m^2^). The 95% CI in genetic analyses are represented by the shaded patterns. BMI= body mass index, CI= confidence limits, CVD= cardiovascular disease, HR= hazard ratio, ICH= intracerebral haemorrhage, IHD= ischaemic heart disease, IS= ischaemic stroke, MVE= major vascular disease

**
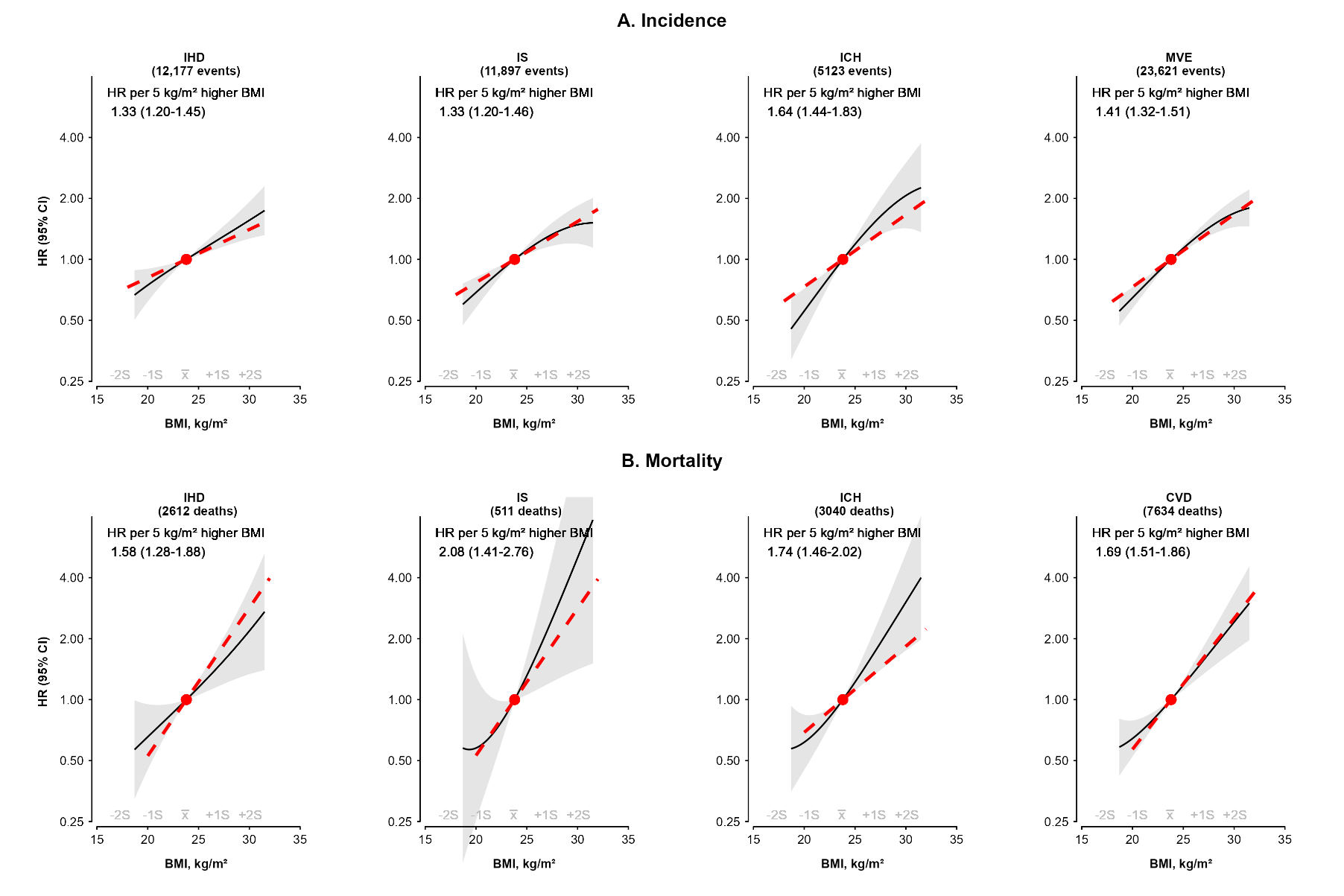
**

# Supplementary references

1. Chen Z, Chen J, Collins R, et al. China Kadoorie Biobank of 0.5 million people: survey methods, baseline characteristics and long-term follow-up. Int J Epidemiol. 2011;**40**:1652-66.

2. Chen Z, Lee L, Chen J, et al. Cohort profile: the Kadoorie Study of Chronic Disease in China (KSCDC). Int J Epidemiol. 2005;**34**:1243-9.

3. Walters RG, Millwood IY, Lin K, et al. Genotyping and population characteristics of the China Kadoorie Biobank. Cell Genom. 2023;**3**:100361.

4. Fairhurst-Hunter Z, Lin K, Millwood IY, et al. Trans-ancestry meta-analysis improves performance of genetic scores for multiple adiposity-related traits in East Asian populations. medRxiv, doi: <https://doi.org/10.1101/2022.07.05.22277254>, 7 July 2022, Preprint: not peer-reviewed.

5. Du H, Bennett D, Li L, et al. Physical activity and sedentary leisure time and their associations with BMI, waist circumference, and percentage body fat in 0.5 million adults: the China Kadoorie Biobank study. Am J Clin Nutr. 2013;**97**:487-96.

6. Easton DF, Peto J, Babiker AG. Floating absolute risk: an alternative to relative risk in survival and case-control analysis avoiding an arbitrary reference group. Stat Med. 1991;**10**:1025-35.

7. Dale CE, Fatemifar G, Palmer TM, et al. Causal Associations of Adiposity and Body Fat Distribution With Coronary Heart Disease, Stroke Subtypes, and Type 2 Diabetes Mellitus: A Mendelian Randomization Analysis. Circulation. 2017;**135**:2373-88.

8. Park JW, Lee SY, Kim SY, Choe H, Jee SH. BMI and stroke risk in Korean women. Obesity (Silver Spring). 2008;**16**:396-401.

9. Song YM, Sung J, Davey Smith G, Ebrahim S. Body mass index and ischemic and hemorrhagic stroke: a prospective study in Korean men. Stroke. 2004;**35**:831-6.

10. Harshfield EL, Georgakis MK, Malik R, Dichgans M, Markus HS. Modifiable Lifestyle Factors and Risk of Stroke: A Mendelian Randomization Analysis. Stroke. 2021;**52**:931-6.

11. Larsson SC, Scott RA, Traylor M, et al. Type 2 diabetes, glucose, insulin, BMI, and ischemic stroke subtypes: Mendelian randomization study. Neurology. 2017;**89**:454-60.

12. Marini S, Merino J, Montgomery BE, et al. Mendelian Randomization Study of Obesity and Cerebrovascular Disease. Ann Neurol. 2020;**87**:516-24.
